# T1-weighted/T2-weighted ratio reflects microstructural changes in Alzheimer’s disease

**DOI:** 10.1186/s13195-026-02093-6

**Published:** 2026-05-27

**Authors:** N. Reijner, M. Riscado Ramos, E. Jacobs, D. V. Toen, B. Tijms, A. J. M. Rozemuller, L. van der Weerd, F. Barkhof, W. D. J. van de Berg, L. E. Jonkman

**Affiliations:** 1 Department of Anatomy and Neurosciences, Section Clinical Neuroanatomy and Biobanking, Amsterdam UMC, De Boelelaan 1108, Amsterdam, 1081 HZ The Netherlands; 2 Amsterdam Neuroscience, Program Neurodegeneration and Brain Imaging, Amsterdam, The Netherlands; 3 Department of Radiology, Leiden UMC, Leiden, The Netherlands; 4 Department of Neurology, Alzheimer Centre Amsterdam, Amsterdam UMC, Amsterdam, The Netherlands; 5 Department of Pathology, Amsterdam UMC, Amsterdam, The Netherlands; 6 Department of Human genetics, Leiden UMC, Leiden, The Netherlands; 7 Department of Radiology and Nuclear Medicine, Amsterdam UMC, Amsterdam, The Netherlands; 8 Institutes of Neurology and Healthcare Engineering, University College London, London, UK

**Keywords:** Imaging, Histology, Pathology, Neurodegeneration, Heterogeneity, Subtype, Phenotype

## Abstract

**Background:**

The T1-weighted/T2-weighted ratio (T1w/T2w-ratio) has been considered as a relatively simple neuroimaging approach to map myelin content in the aging and diseased brain. However, we hypothesize that any process affecting the tissue microstructural integrity also contributes to observed changes in the T1w/T2w-ratio. Here, we aim to evaluate the ability of T1w/T2w-ratio to detect microstructural changes, specifically within the paradigm of Alzheimer’s disease (AD).

**Methods:**

Postmortem in-situ T1-weighted and T2-weighted MRI scans of 27 control and 51 AD brain donors were processed into standardized T1w/T2w-ratio images and parcellated into cortical regions according to the brainnetome atlas. Using an identical pipeline, antemortem MRI scans of 9 AD cases were processed. Immunohistochemical staining and digital quantification were performed for amyloid-beta (4G8), phosphorylated tau (AT8), neuro-axonal damage (NfL), myelin (PLP), microglia (IBA1), and iron (Meguro) in 9 cortical regions. Data was analysed using linear mixed models.

**Results:**

Whole cortex T1w/T2w-ratio was 15% lower in AD compared to controls and was 13% lower in the hippocampus of typical AD compared to atypical AD clinical phenotypes. Additionally, a strong correlation was found between antemortem and postmortem T1w/T2w-ratios (*r* = 0.85). Whole-brain associations between T1w/T2w-ratio and histological changes were found in both control and AD patients: T1w/T2w-ratio associated with myelin density (Controls: β = 0.229; AD: β = 0.312) and microglia density (Controls: β=-0.090; AD: β=-0.496). In AD but not in controls, T1w/T2w-ratio associated with amyloid-beta (β = 0.233), phosphorylated tau (β=-0.220), and neuro-axonal damage (β = 0.170). Iron load showed contrasting associations in controls and AD (Controls: β=-0.359; AD: β = 0.154).

**Conclusion:**

The findings in this study suggest that the T1w/T2w-ratio is mainly associated with myelin density in healthy controls, but is a broader indicator of microstructural integrity in Alzheimer’s disease, where it additionally largely associates with accumulation of amyloid-beta, phosphorylated tau and neuro-axonal damage. Therefore, the T1w/T2w-ratio is not a myelin-specific marker in AD, but could be a valuable neuroimaging tool for tracking longitudinal microstructural changes when interpreted alongside established AD pathology biomarkers.

**Supplementary Information:**

The online version contains supplementary material available at 10.1186/s13195-026-02093-6.

## Background

The pathological hallmark of Alzheimer’s disease (AD) is the aggregation of amyloid-beta (Aβ) into plaques and phosphorylated tau (pTau) into neurofibrillary tangles (NFTs), leading to neurodegeneration and cognitive impairment [1]. Additionally, myelin disruption, manifesting as loss or disorganization of the myelin sheath, has been shown to be a prominent feature in AD, potentially even preceding Aβ and pTau aggregation [2, 3]. Non-invasive assessment of anatomical patterns of myelin integrity in the brain can only be performed indirectly using MRI approaches, such as diffusion tensor imaging, multi-compartment water fraction techniques, and quantitative susceptibility mapping [4–6]. An alternative to these approaches leverages the T1-weighted and T2-weighted MRI sequences, which are both routinely acquired in clinical care settings as part of standard structural imaging protocols, where a ratio between the sequences (T1w/T2w-ratio) is used to assess myelin integrity [7].

Previous studies suggest that the T1w/T2w-ratio provides a reliable neuroimaging marker for mapping and monitoring cerebral myelin content in healthy brains, and sensitively detects white matter lesions, indicative of extensive myelin damage, in patients with multiple sclerosis (MS) [8–11]. When applied in AD, previous studies found the T1w/T2w-ratio to be altered in cortical brain regions compared to controls [12–16]. Although findings vary, most of these studies observed a decrease in T1w/T2w-ratio, predominantly in regions typically vulnerable to AD (hippocampus, temporal lobe, precuneus), as well as an association with overall cognitive decline. Interestingly, several AD-focused studies found the T1w/T2w-ratio also associated with Aβ and pTau aggregation (CSF and PET measured), suggesting a sensitivity beyond myelin integrity [14, 17, 18]. Notably, in healthy individuals, the T1w/T2w-ratio aligns more consistently with myelin integrity, possibly because of the absent interference from pathological processes [4, 10, 19]. Aberrant pathological processes such as protein accumulation, inflammation, iron accumulation and neurite density loss can influence either T1-weighted or T2-weighted signals, and consequently the T1w/T2w-ratio [14, 20, 21]. This suggests the T1w/T2w-ratio may reflect other processes that change the general microstructural integrity of brain tissue, rather than myelin alone [22, 23]. This proposed ability of the T1w/T2w-ratio to reflect microstructural integrity has yet to be validated using an approach that directly and comprehensively measures microstructural changes across the cortex.

Additionally, conventional neuroimaging still struggles to capture disease heterogeneity, where this broader sensitivity of the T1w/T2w-ratio may offer an advantage. For instance, atypical (i.e. non-amnestic) AD clinical phenotypes differ from typical (i.e. amnestic) AD clinical phenotypes in regional atrophy patterns, driven by microstructural integrity patterns, such as regional pTau aggregation [24, 25]. Unfortunately, atrophy patterns alone show low sensitivity for distinguishing phenotypes, partly due to overlapping regions that blur diagnostic boundaries [26]. The T1w/T2w-ratio may improve sensitivity to microstructural integrity differences, potentially capturing the more cortically dominant changes expected in atypical AD [25]. While currently underexplored, such non-conventional neuroimaging approaches could be valuable for disentangling AD heterogeneity in both research and clinical settings.

The hypothesis of the current study is that the T1w/T2w-ratio predominantly maps myelin integrity in healthy brain tissue but is a reflection of more widespread microstructural integrity changes in pathologically burdened brain tissue. This is investigated using a unique postmortem cohort consisting of both control and AD brain donors, where both postmortem in-situ (brain still in cranium) MRI data and histological data are utilized. The association between cortical T1w/T2w-ratio measurements and histological markers including Aβ and pTau aggregation, neuro-axonal damage, myelin, iron accumulation, and microglia-mediated inflammation will be investigated on a whole-brain as well as a region-specific level. The observations of this study will provide a more comprehensive answer to how the T1w/T2w-ratio reflects microstructural change, particularly in pathologically burdened brains, such as seen in AD.

## Materials and methods

### Donor inclusion

In collaboration with the Netherlands Brain Bank (NBB; http://brainbank.nl*)* we included 52 Alzheimer’s disease donors, 40 of whom were from the Amsterdam Dementia Cohort [27]. Of these Alzheimer’s disease donors, 25 were diagnosed as typical (amnestic) and 20 as atypical (non-amnestic) Alzheimer’s disease on the basis of initial dominant clinical symptoms [25]. Neuropathological diagnosis was confirmed, and concomitant pathologies were identified, by an expert neuropathologist (A.J.M.R.) and performed according to the international guidelines of the Brain Net Europe II (BNE) consortium [28, 29]. This includes assessment of cytoarchitecture abnormalities, and pathological scoring of amyloid (Thal phases) [30], cerebral amyloid angiopathy (CAA) [31], pTau (Braak neurofibrillary tangles (NFT) stages) [32], alpha-synuclein (Braak aSyn staging) [33, 34] and TAR DNA-binding protein 43 (TDP-43)(limbic-predominant age-related TDP-43 encephalopathy neuropathologic changes (LATE-NC) staging) [35]. Additionally, 27 age and sex-matched non-neurological controls were selected from the Normal Aging Brain Collection Amsterdam (NABCA; http://nabca.eu*)* [36]. All control donors had an AD neuropathological change score of none/low and no extensive co-pathologies [37]. All donors signed an informed consent for brain donation, and the use of material and clinical information for research purposes. The procedures for brain tissue collection of NBB and NABCA have been approved by the Medical Ethical Committee of Amsterdam UMC (formerly known as VUmc). All donors had MRI data available but tissue for histological quantification was available for a subset; 36 AD donors and 17 control donors. See supplementary Table 1 for a detailed list of included donors.

### Postmortem in-situ and antemortem in vivo MRI acquisition

Postmortem 3T in-situ (brain in cranium) MRI scans were acquired according to a previously described pipeline [36]. Briefly, the following 3T scans (Signa-MR750, General Electric Medical Systems, United States) were acquired with an eight-channel phased-array head-coil: (i) a sagittal 3D T1-weighted fast spoiled gradient echo sequence [repetition time (TR) = 7 ms, echo time (TE) = 3 ms, flip angle = 15°, 1-mm-thick axial slices, in-plane resolution = 0.5 × 0.5 mm^2^] (ii) an axial 2D T2-weighted turbo spin echo sequence [repetition time (TR) = 4246 ms, echo time (TE) = 114 ms, flip angle = 111°, 3-mm-thick axial slices, in-plane resolution = 0.5 × 0.5 mm^2^]. Moreover, 9 Alzheimer’s disease cases included in our study had clinical antemortem 3T T1 and T2 MRI scans available within 2 years before death. See supplementary Table 2 for antemortem MRI acquisition details. Acquired scans were assessed for abnormalities and visual atrophy scoring by an expert radiologist (F.B.).

### Calculation of T1-weighted/T2-weighted ratio and standardization

T1-weighted and T2-weighted MR images were analysed according to the methodology set out by Glasser and Van Essen [7]. The Statistical Parametric Mapping Software version 12 (SPM12) running in Matlab (version 2022b) was used for processing. Tissue segmentation of grey matter (GM), white matter (WM), and cerebrospinal fluid (CSF), was applied to T1-weighted images to produce GM probability maps. A probability threshold of 0.3 was applied to GM probability maps in an attempt to limit partial volume effects. Additionally, a sensitivity analysis was performed to show results are similar across GM probability thresholds (Supplementary Fig. 1). T2-weighted images were co-registered to T1-weighted images and both images were bias field corrected. For each subject, the brainnetome atlas [38] was used to parcellate the brain into 210 cortical regions, which were warped to subject-specific space using inverted deformation fields generated by SPM12, creating subject-specific GM atlases. Additionally, these atlases were used to extract regional volumes. To standardize the T1w/T2w-ratio, the method described by Ganzetti et al. [39] was used. In short: subject-specific eye and the temporalis muscle masks were generated using the ITK-SNAP (version 3.8.0) [40] semi-automatic segmentation algorithm, the mode signal intensity of these structures was calculated, and a linear scaling formula was applied to normalize subject image intensity relative to the MNI152 template image of the same modality. Finally, standardized T1-weighted images were divided by standardized T2-weighted images and masked with the subject-specific GM atlases to obtain standardized T1w/T2w-ratio means for each region. All images were visually inspected for errors, only one case had to be excluded due to inability to generate standardization masks. For a flow diagram of the applied pipeline see supplementary Fig. 2.

### Tissue sampling

After MRI acquisition, autopsy was performed, all within 12 h after death. Fixed right brain hemispheres (four weeks in 4% buffered PFA) were cut into 1 cm slices and dissected into anatomically defined tissue blocks by a neuropathologist (A.J.M.R.) for the NBB AD cases, and by a neuroanatomist (W.v.d.B.) for the NABCA control cases, according to the protocol of BrainNET Europe [41]. Tissue was subsequently paraffin-embedded as previously described [36]. The following regions of the right hemisphere were included for the current study: middle frontal gyrus (GFM), middle temporal gyrus (GTM), superior parietal gyrus (GPS), posterior cingulate gyrus (PCC), precuneus (Precun), primary visual cortex of the occipital cortex (OC), hippocampus (tissue block includes Hip (Dentate gyrus, CA1-4, subiculum), Parahippocampus (entorhinal and parahippocampal gyri) and fusiform gyrus).

### Immunohistochemistry

6 µm sections from the above-mentioned regions were cut and mounted on superfrost+ glass slides (Thermo Scientific USA). Sections were stained for amyloid-beta (clone 4G8, Biolegend, 1:8000), phosphorylated tau (clone AT8, Thermo Scientific, 1:800), neurofilament light-chain (NFL, Synaptic Systems 1:600), Myelin (PLP, Bio-Rad, 1:500) and Microglia (IBA1, Wako Chemicals 1:1000). Sections underwent heat induced epitope retrieval (HIER) for 30 minutes in a steamer in pre-heated 10 mM Citrate Buffer pH 6.0 (4G8, AT8 and IBA1) or 10 mM Tris-EDTA buffer pH 9.0 (NfL and PLP). The sections were blocked for endogenous peroxidase using 0.3% hydrogen peroxide in tris buffered saline (TBS; pH 7.6) and subsequently blocked for non-specific binding using 3% normal goat serum and 0.5% Triton X. Primary antibodies were diluted in blocking serum and incubated overnight at 4°C. Primary antibodies were detected using EnVision (Dako, Glostrup, Denmark), and visualized using 3,3’-Diaminobenzidine (DAB, Dako) with Imidazole (50 mg DAB, 350 mg Imidazole and 1 µl of H2O2 per 100 ml of Tris–HCl 30 mM, pH 7.6). Sections were counterstained with haematoxylin. Between steps, TBS was used to wash the sections. For iron visualization, 20 μm sections were cut, mounted on superfrost+ glass slides (Thermo Scientific USA), and stained within 5 days with a modified Meguro staining protocol [42]. Briefly, sections were incubated in 1% potassium ferrocyanide solution. Followed by incubation in methanol solution containing 0.01% NaN_3_ and 0.3% H_2_O_2_ for enhancement of subsequent DAB staining (25 mg DAB and 17 µl of H2O2 per 100 ml of phosphate buffer; pH 7.4). Between steps, sections were washed with phosphate buffer. For all staining protocols, sections were dehydrated and mounted with Entellan (Merck, Darmstadt, Germany) after completion.

### Histological quantification

Images were taken using a whole-slide scanner (Vectra Polaris, 20× objective) and quantified using QuPath Version 5.1 (https://qupath.github.io/). Regions of interest containing all cortical layers were delineated in straight areas of the cortex, to avoid over- or underestimation of pathology in sulci and gyri, respectively [43]. Hippocampus sections were segmented into hippocampus proper (including dentate gyrus, cornu ammonis (CA) 1–4, subiculum and parasubiculum) and parahippocampal regions (entorhinal cortex and parahippocampal gyrus combined) according to previously described anatomical characteristics, and to match MRI annotated regions [44, 45].

Using QuPath’s pixel classifier function, a machine learning model was trained on a representative subset of the data to quantify the DAB positive signal. For each immunohistochemical (IHC) marker, the outcome measure was the % of immunoreactivity per area of interest (area%) for each brain region. In summary, the IHC outcome measures were area% of Aβ, pTau, NfL, PLP, IBA1 and Iron (see supplementary Fig. 3).

### Microglia morphological analysis pipeline

In order to quantify microglia mediated inflammation, microglia were classified into ramified (non-inflammatory), reactive (inflammatory) or ameboid (inflammatory), using morphological characteristics extracted using a custom setup of an automatic morphological quantification pipeline. To achieve this, microglia were first isolated by creating a machine learning approach using Qupath’s pixel classifier function in combination with its object classifier function. Per region of interest, 20 microglia were selected at random resulting in over 20.000 isolated microglia images, which were further processed in FIJI [46]. Here, microglia images were optimized for maximum contrast and minimum noise. Microglia soma size was measured, microglia were skeletonized for branch analysis using the “Analyze Skeleton” plugin [47], followed by a Sholl analysis, using the “Sholl analysis” plugin [48], to measure the ramification index. Finally the “FracLac” plugin was used for fractal dimension and lacunarity analysis [49]. Microglia were then classified into inflammatory and non-inflammatory using manual classification (for ameboid morphology) and k-means clustering (for reactive and ramified morphologies). An inflammation ratio was generated by calculating the ratio between inflammatory and non-inflammatory morphological classifications where ( $Inflammation=\frac{Ameboid+Reactive}{Ramified}$), resulting in a higher score indicating more relative inflammation. See supplementary Fig. 4 for a more detailed overview of the microglia morphological analysis pipeline.

### Statistical analysis

All statistical analyses were performed in Rstudio Version 4.3.2. Cohort characteristics were analysed using linear regression models for continuous data, and Fisher’s exact test for categorical data. Group differences were analysed using linear mixed models with T1w/T2w-ratio or a histological marker as dependent variable, grouping variable as independent variable and random intercepts for subjects when analysing global (regions combined) effects, region was added as an interaction term when analysing regional effects. A post-hoc analysis was then performed using the estimated marginal means generated by the model in order to test group differences. Because of between-batch immunostaining differences present in the PLP staining, batch corrections were applied using the “batchtma” R package which performed a marginal standardization, reducing between-batch variation while retaining in-batch variation.

Associations between T1w/T2w-ratio and immunohistological findings were assessed using linear mixed models with histological marker of interest as independent variable. Two complementary models were defined to assess whole cortex associations.

#### Model 1 (between-region model)

$$T1w/T2w-ratio=HistoMarker\star\:Diagnostic\:Group+Covariates+\left(1\mid{Subject}\right)$$

This model includes subject as random intercept, accounting for individual differences in overall T1w/T2w-ratio levels. It isolates between-region differences in T1w/T2w-ratio, capturing effects associated with patterns across the brain. This model provides sensitivity to explain variations in whole-brain T1w/T2w-ratio on a within-subject level such that it can assess whether, within each subject, regions with higher marker levels have higher T1w/T2w-ratio.

#### Model 2 (within-region model)

$$T1w/T2w-ratio=HistoMarker\star\:Diagnostic\:Group+Covariates+\left(1\mid{Subject}\right)+\left(1\mid{Region}\right)$$

This model additionally introduces region as random intercept, accounting for differences between regions. This more conservative model isolates within-region differences in T1w/T2w-ratio, capturing whole-brain effects that are consistent within each region across subjects.

Additionally, regional effects were assessed by adding region as interaction term with the histological marker of interested in model 1. A post-hoc analysis using estimated marginal means was used to retrieve regression coefficients. Standardized regression coefficients (βs) were calculated by standardizing input variables, improving interpretability by allowing a one-unit increase in a variable to be equal to its standard deviation. Note that control group T1w/T2w-ratio associations with pTau have been omitted as by definition this group is limited to Braak Stage ≤ 3 and has no neocortical pTau burden.

Correlations between antemortem and postmortem T1w/T2w-ratios were tested using repeated-measures correlations (“rmcorr” R package). Correlations between immunohistological markers was tested using Spearman’s correlations.

A *p*-value of ≤ 0.05 was considered significant throughout all analyses and in all analyses multiple comparison adjustment using the false discovery rate (FDR) method was performed. Analyses including only histological data were corrected for age at death and sex; analyses including MRI data were corrected for age at death, sex, postmortem delay (PMD) and total grey matter volume. Percent difference between groups was calculated as: percent difference = |Value 1 – Value 2|/((Value1 + Value 2)/2) * 100.

## Results

### Cohort demographics

Control and AD groups showed no significant differences in sex, age at death, and postmortem delay (PMD) (all *p* > 0.05). There was a higher prevalence of APOE4 gene carriers in the AD group compared to controls (Controls: 29.2% vs. AD: 69.2%, *p* = 0.006) (see Table 1). Compared to controls, AD cases had lower normalized whole brain volume (-14.4%, *p* < 0.001), normalized grey matter volume (-15.5%, *p* < 0.001), and normalized white matter volume (-12%, *p* < 0.001). Compared to controls, AD cases had higher visual scores for global cortical atrophy (*p* < 0.001), parietal cortical atrophy (*p* < 0.001), medial temporal lobe atrophy (*p* < 0.001) and Fazekas (*p* = 0.008). As per definition, pathology scores were higher in AD compared to controls for Braak neurofibrillary tangle (NFT) stage (*p* < 0.001), Thal amyloid-beta (Aβ) phase (*p* < 0.001), LATE stage (*p* = 0.010) and CAA-type (*p* < 0.001), with CAA being absent for most control cases. No differences were found for Braak aSyn stage (*p* = 0.40).

**Table 1:** Cohort characteristics

	Control	AD	Typical AD	Atypical AD
	*n* = 27	*n* = 51	*n* = 25 (out of 51)	*n* = 20 (out of 51)
Sex (M/F)	14/13 (52%/48%)	34/17 (67%/33%)	19/6 (76%/24%)	12/8 (60%/40%)
Age at death (In years)	73 (± 8) [57–87]	69 (± 10) [37–89]	70 (± 11) [53–89]	65 (± 10) [37–78]
Age at Onset (In years)	NA	63 (± 10) [32–81]	63 (± 10) [46–81]	60 (± 9) [32–74]
Disease duration (In years)	NA	6 (± 4) [1–14]	7 (± 4) [1–14]	4 (± 3) ^##^ [1–10]
Clinical Subtyping Typical/Atypical	NA	25/20 NA = 6	25/0	0/20
APOE genotyping APOE4 carrier	7 (29.2%) NA = 3	27 (69.2%)** NA = 12	13 (65%) NA = 5	12 (70.6%) NA = 3
Postmortem delay (Hours: minutes)	8:38 (± 2:21)	6:45 (± 1:37)	8:02 (± 1:25)	7:56 (± 1:43)
MRI				
Normalized Whole Brain Volume (% ICV)	70.0 (± 4.4)	60.6 (± 6.4)***	60.5 (± 6.2)	60.8 (± 7.4)
Normalized Gray Matter Volume (% ICV)	40.4 (± 3.2)	34.6 (± 4.6)***	35.2 (± 4.8)	34.2 (± 5.0)
Normalized White Matter Volume (% ICV)	29.6 (± 2.6)	26.0 (± 4.6)***	25.3 (± 5.4)	26.6 (± 3.9)
Global Cortical Atrophy 0/1/2/3/4	13/3/0/0/0 NA = 11	7/16/8/6/1*** NA = 13	2/10/5/2/1 NA = 5	5/6/2/4/0 NA = 3
Parietal Cortical Atrophy 0/1/2/3	10/5/1/0 NA = 11	1/15/16/8*** NA = 11	0/9/7/3 NA = 6	1/4/9/5 NA = 1
Medial Temporal Lobe Atrophy 0/1/2/3/4	11/12/4/0/0	4/13/12/11/11***	2/6/7/4/6	2/6/4/6/2
Fazekas 0/1/2/3	13/5/9/0	16/11/10/14**	8/6/3/8	7/5/6/3
Pathology				
Braak NFT stage 0/1/2/3/4/5/6	4/15/8/0/0/0/0	0/0/0/0/5/18/28 ***	0/0/0/0/4/9/12	0/0/0/0/1/7/12
Thal Aβ phase 0/1/2/3/4/5	5/12/6/4/0/0	0/0/0/2/2/47 ***	0/0/0/2/0/23	0/0/0/0/2/18
Braak aSyn stage 0/1/2/3/4/5/6 Amygdala predom	22/2/1/2/0/0 0	39/1/1/1/1/2/1 4	20/1/1/0/0/0/0 3	15/0/0/1/1/2/0 1
LATE stage 0/1/2/3	27/0/0/0	36/5/8/2 *	17/4/3/1	16/1/2/1
CAA-Type 0/1/2	19/2/6	1/34/16 ***	1/21/3	0/10/10^##^

Data is noted as mean (± standard deviation) [range] or count (ratio %). Significance between control and AD groups is denoted with * = *p* ≤ 0.05, ** = *p* ≤ 0.01, *** = *p* ≤ 0.001. Significance between typical AD and atypical AD groups is denoted with # = *p* ≤ 0.05, ## = *p* ≤ 0.01, ### = *p* ≤ 0.001. *NA* not available, *ICV*  Intracranial Volume, *NFT*  Neurofibrillary Tangle, *A*β  Amyloid-beta, *aSyn*  alpha-synucleinopathy, *LATE*  Limbic-predominant Age-related TDP-43 Encephalopathy, *CAA*  Cerebral Amyloid Angiopathy

Group comparisons between clinical AD phenotypes showed a lower disease duration in atypical AD cases (typical AD: 7 years vs. atypical AD: 4 years, *p* = 0.009). While age of onset was lower in atypical AD compared to typical AD (typical AD:63, vs. atypical AD:60 years, *p* = 0.296), this difference was not significant. Additionally, a difference in CAA pathology was observed with CAA-type 1 being more prevalent in typical AD and CAA-type 2 being more prevalent in atypical AD (*p* = 0.008).

See supplementary Table 3 for demographics of the subset of cases for which histological data is available.

### T1w/T2w-ratio is decreased in AD

**Fig. 1:**
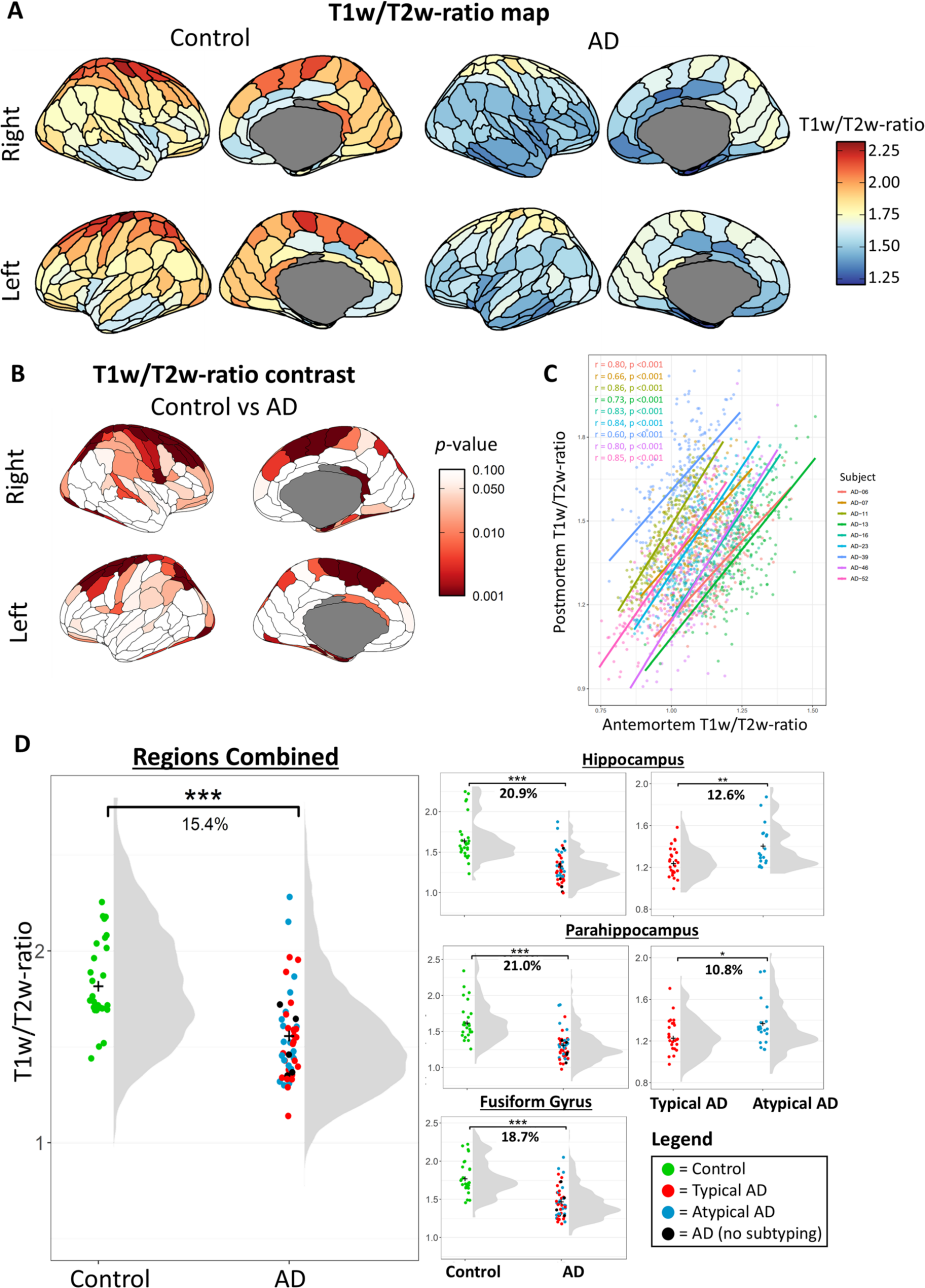
T1w/T2w-ratio distribution and contrast between groups. **A** Regional distribution map of the T1w/T2w-ratio for control and AD groups. **B** Brain map showing regions with significant cortical contrast differences (*p*-values) between controls and AD, highlighting a regional decrease in the T1w/T2w-ratio in AD. **C** Pearson correlation analysis between postmortem and antemortem for each subject (within 2 years of death), with datapoints representing cortical regions. Correlation coefficients of T1w/T2w-ratios show a strong correlation between post- and antemortem measurements (mean *r* = 0.77). **D** Global and regional distribution of T1w/T2w-ratios in controls, AD, and AD clinical phenotypes. A global reduction in the T1w/T2w-ratio is observed in AD (15.4%), with the most prominent decrease in the medial temporal lobe. Significant regional differences are also noted between AD clinical phenotypes. Points represent mean values for each subject; raincloud plots illustrate the overall distribution of regional datapoints. * = *p* ≤ 0.05, ** = *p* ≤ 0.01, *** = *p* ≤ 0.001, ns = not significant, + = estimated marginal mean, AD = Alzheimer’s Disease

Figure 1A illustrates normal T1w/T2w-ratio mapping, where the control group shows a pattern of highest T1w/T2w-ratio (> 2.0) in pre- and postcentral regions, high T1w/T2w-ratio (> 1.8) in prefrontal and occipital regions and in the posterior cingulate, and lowest T1w/T2w-ratio (< 1.5) in the inferior frontal and temporal regions.

Global (regions combined) T1w/T2w-ratio was lower in AD compared to controls (15.4%, *p =* 0.001), this difference was most pronounced in the hippocampus (20.9%, *p* < 0.001), parahippocampus (21%, *p* < 0.001) and fusiform gyrus (18.7%, *p* < 0.001)(see Fig. 1B, D). Typical AD cases showed a lower T1w/T2w-ratio compared to atypical AD cases in both the hippocampus (12.6%, *p* = 0.005) and parahippocampus (10.8%, *p* = 0.028) (Fig. 1D).

To investigate a possible left-right asymmetry in T1w/T2w-ratio values, we directly compared left and right hemisphere regions. In the control group, little to no differences between hemispheres were observed. While in AD a lower T1w/T2w-ratio was observed in the right hemisphere, most pronounced in frontal, parietal, and temporal regions (supplementary Fig. 5).

To investigate the agreement between postmortem and antemortem measured T1w/T2w-ratio of a subject, a Pearson correlation analysis was performed on subjects with a postmortem to antemortem interval within 2 years. Correlation ranged from moderate to strong (range: *r* = 0.6 – *r* = 0.86, all *p* < 0.001) with a mean correlation coefficient of *r* = 0.77 (Fig. 1C). Additionally, using linear mixed models, adjusted for age, sex, and total grey matter volume with subject and region as random effects, an association estimate of β = 0.87 (*p* < 0.001) between postmortem and antemortem T1w/T2w-ratio values was found. Adding APOE status as covariate did not change this effect.

When only assessing the nine right hemisphere regions included for IHC instead of all atlas regions, a similar cross-regional and regional decrease in T1w/T2w-ratio was found (supplementary Fig. 6).

Finally, we investigated potential effects of APOE4 on the T1w/T2w-ratio measurements. Group comparison of global T1w/T2w-ratio between APOE4 carriers and non-carriers showed there was no significant difference between groups. Additionally, APOE4 contribution as covariate to T1w/T2w-ratio group comparisons between control vs. AD as well as between typical vs. atypical AD was found to be non-significant (Supplementary Fig. 7).

### AD brains show extensive microstructural changes

**Fig. 2:**
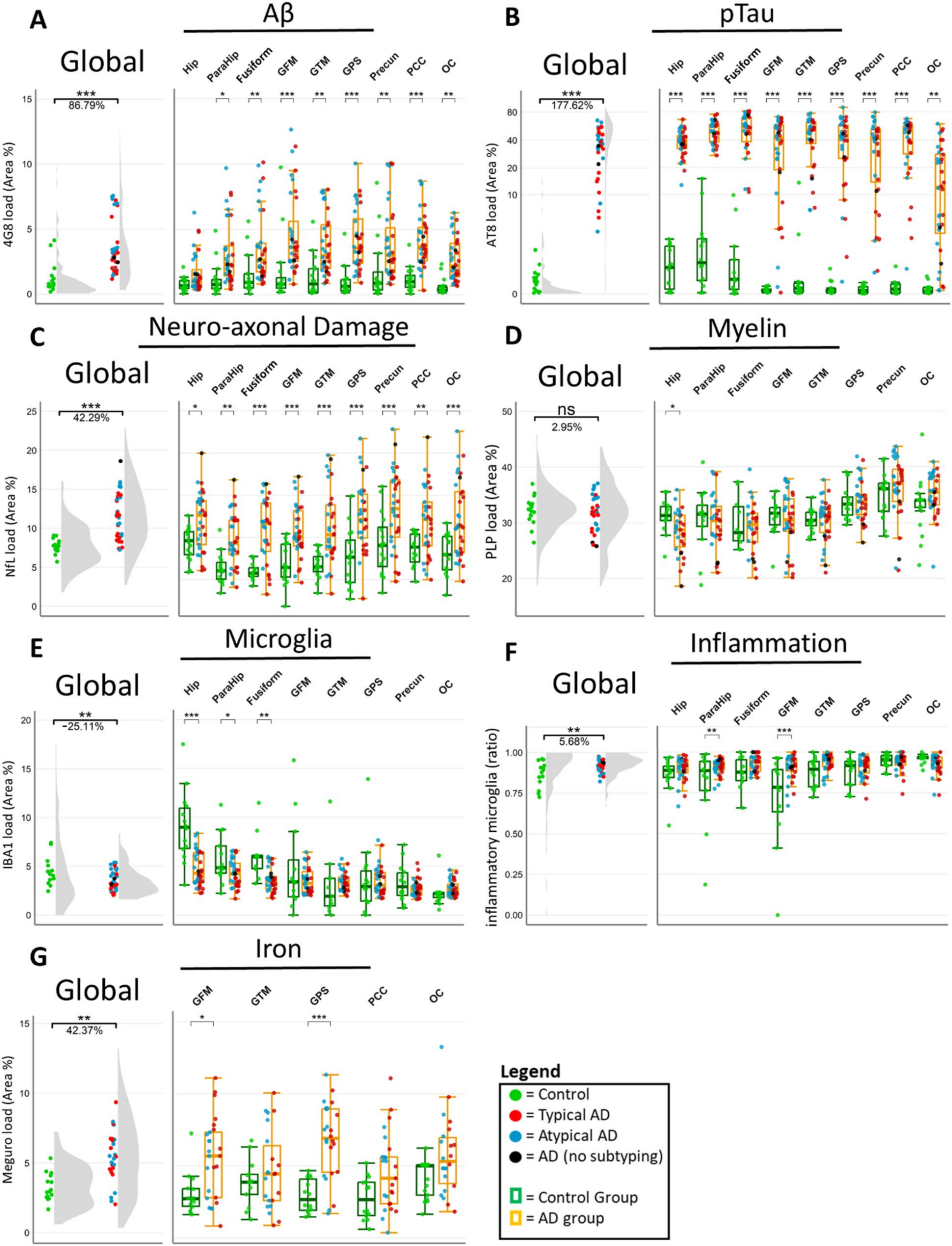
Regions combined and regional distribution of histological markers. **A** Aβ (4G8) distribution patterns showed an overall increase in AD compared to controls (86.79%) and in all regions except for the hippocampus. **B** pTau (AT8) distribution showed an overall increase in AD compared to controls (177.62%) and in all regions (y-axis is logarithmically scaled). **C** Neuro-axonal (NfL) damage distribution showed an overall increase in AD compared to controls (42.29%) and in all regions, except for the hippocampus. **D** Myelin (PLP) density distribution only shows to be decreased in the hippocampus of AD when compared to controls. **E** Microglia (IBA1) density distribution shows an overall decrease in AD compared to controls (-25.11%), predominantly in the hippocampal, parahippocampal and fusiform regions. **F** ratio between inflammatory-and-non-inflammatory microglial morphology phenotype measured inflammation distribution showed an overall increase in AD compared to controls (5.68%), predominantly in the parahippocampus and middle frontal gyrus. **G** Iron (Meguro) accumulation distribution shows an overall increase in AD compared to controls (43.37%), predominantly in the middle frontal and superior parietal gyri. Points represent mean values for each subject; raincloud plots illustrate the overall distribution of regional datapoints. Hip = hippocampus, ParaHip = parahippocampal gyrus, GFM = middle frontal gyrus, GTM = middle temporal gyrus, GPS = superior parietal gyrus, Precun = precuneus, PCC = posterior cingulate cortex, OC = occipital cortex. * = *p* ≤ 0.05, ** = *p* ≤ 0.01, *** = *p* ≤ 0.001, ns = not significant, + = estimated marginal mean, AD = Alzheimer’s Disease

An overall (regions combined) increase in immunoreactivity for Aβ (86.8%, *p* < 0.001), pTau (177.6%, *p* < 0.001) and neuro-axonal damage (42.3%, *p* < 0.001) was found in AD cases compared to controls. Similarly, within all regions, an increase in Aβ, pTau and neuro-axonal damage was found in AD compared to controls, except for Aβ in the hippocampus (Fig. 2A-C). Myelin density did not show any differences globally, regionally the hippocampus showed a lower myelin density in AD compared to controls (-13.1%, *p* = 0.036) (Fig. 2D). For microglia density an overall (regions combined) decrease was observed in AD compared to controls (-25.1%, *p* = 0.010), as well as a decrease in medial temporal lobe regions: hippocampus (-64.27%, *p* < 0.001), parahippocampus (-31.1%, *p* = 0.003) and fusiform gyrus (-48%, *p* = 0.030) (Fig. 2E). Inflammation (defined as the ratio between inflammatory and non-inflammatory microglia) was increased in AD compared to controls (5.7%, *p* = 0.005), this was most pronounced in the parahippocampus (12.2%, *p* = 0.002) and the middle frontal gyrus (22.9%, *p <* 0.001), see Fig. 2F. Iron accumulation showed an overall (regions combined) increase in AD compared to controls (42.4%, *p* = 0.001), most pronounced in the middle frontal gyrus (52%, *p* = 0.01) and the superior parietal gyrus (74%, *p* < 0.001)(Fig. 2G).

Investigating differences between typical and atypical clinical AD phenotypes, pTau was higher in the precuneus of the atypical AD group (43.9%, *p* = 0.019), and NfL was observed to be higher in the superior parietal gyrus of the atypical AD group, but this difference did not survive multiple comparison adjustment (22.8%, *p* = 0.074) (supplementary Table 4).

For an overview of significant correlations between histological markers, see supplementary Fig. 8.

### T1w/T2w-ratio reflects mainly myelin in controls, but overall microstructural change in AD

**Fig. 3:**
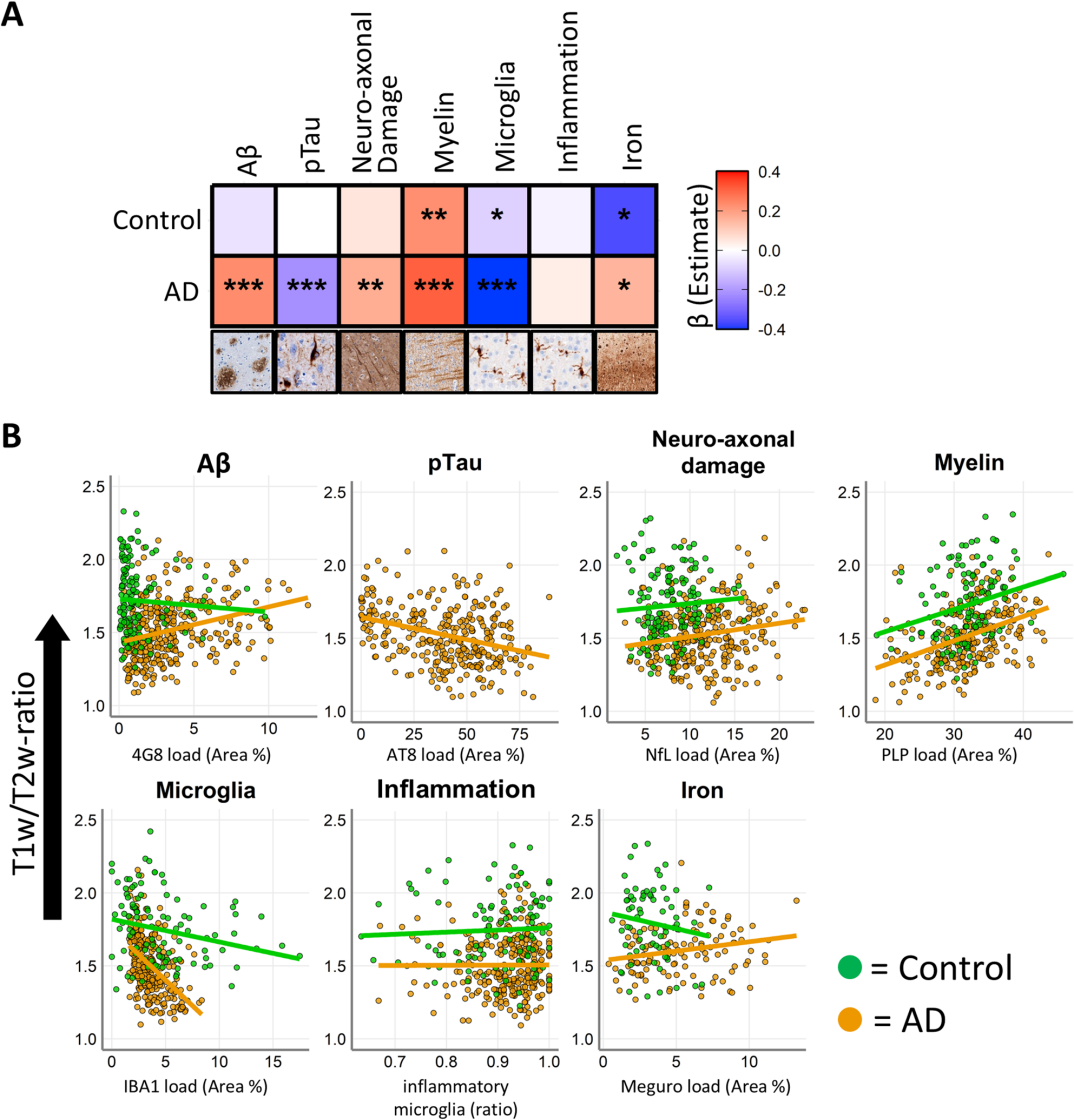
Associations between T1w/T2w-ratio and histological markers. **A** heatmap of associations (β estimate; standardized) for each histological marker in both control and AD groups, showing associations with microstructural changes. **B** Scatterplot of covariate adjusted T1w/T2w-ratio and pathological marker values with estimated marginal regression slopes. Control group associations for pTau have been omitted as by definition this group is limited to Braak Stage ≤ 3 and has no neocortical pTau burden. * = *p* ≤ 0.05, ** = *p* ≤ 0.01, *** = *p* ≤ 0.001

Using the between-region model (Model 1), which accounts for individual differences and captures how T1w/T2w varies across brain regions within subjects, whole-brain positive associations between T1w/T2w-ratio and myelin were found for both control (β = 0.229, *p* = 0.009) and AD (β = 0.312, *p* < 0.001) groups. Additionally, AD cases showed whole-brain associations between T1w/T2w-ratio and Aβ accumulation (β = 0.233, *p* < 0.001), pTau accumulation (β = -0.220, *p* < 0.001) and neuro-axonal damage (β = 0.170, *p* = 0.003) (Fig. 3). Iron accumulation showed a contrasting association between groups, negative in controls (β = -0.359, *p* < 0.001) and positive in AD (β = 0.154, *p* = 0.034). Microglia density showed negative associations with T1w/T2w-ratio in controls (β = -0.090, *p* = 0.044) but showed a stronger association in AD (β = -0.496, *p* < 0.001), no associations were found with microglia mediated inflammation (see supplementary Table 5 for model details).

The potential effect of APOE genotyping on these results was assessed by repeating the between-region models with APOE as additional covariate, which resulted in no changes compared to the main findings (supplementary Table 6).

When assessing associations with the within-region model (Model 2), which isolates whole-brain associations that are consistent within all regions, most associations disappeared. Only the association between T1w/T2w-ratio and iron in controls remained significant (β = -0.257, *p* = 0.031) but did not survive multiple comparison correction.

When analysing regional effects, in AD T1w/T2w-ratio showed to be associated with Aβ accumulation (β = 0.264, *p* = 0.007) and pTau accumulation (β = 0.278, *p* = 0.007) in the occipital cortex, with myelin density in the hippocampus (β = 0.181, *p* = 0.033) and with microglia density in the middle frontal gyrus (β = -0.168, *p* = 0.039). In controls T1w/T2w-ratio associated with Aβ accumulation in the middle frontal gyrus (β = -0.179, *p* = 0.039), with iron in the superior parietal gyrus (β = -0.364, *p* = 0.007) and with microglia density in the parahippocampal region (β = 0.244, *p* = 0.002). Only the association with microglia density in control parahippocampal region survived multiple comparison adjustment (*p* = 0.029) (supplementary Table 7). For additional visualization of regional and regional average associations per histological marker see supplementary Fig. 9.

When investigating the different associations in clinical AD phenotypes, using the between-region model (Model 1), similar associations for Aβ (TypAD: β = 0.320, *p* = 0.001; AtypAD: β = 0.207, *p* = 0.036), myelin density (TypAD: β = 0.336, *p* < 0.001; AtypAD: β = 0.291, *p* < 0.001) and microglia density (TypAD: β = -0.692, *p* < 0.001; AtypAD: β = -0.482, *p* < 0.001) were found. Similarly, no associations with iron accumulation or inflammation for either phenotype was found. (supplementary Fig. 10 and supplementary Table 5). However, T1w/T2w-ratio only associated with pTau in typical AD (β = -0.335, *p* = 0.004), and only with neuro-axonal damage in atypical AD (β = 0.204, *p* = 0.026). No significant associations were found using the within-region model (Model 2). Regional analysis showed an association with Aβ accumulation (TypAD: β = 0.293, *p* = 0.007; AtypAD: β = 0.241, *p* = 0.030), and with pTau accumulation both in the occipital cortex (TypAD: β = 0.257, *p* = 0.030; AtypAD: β = 0.298, *p* = 0.007), none of which survived multiple comparison correction (supplementary Table 5).

## Discussion

This study demonstrates a decreased T1w/T2w-ratio in AD compared to controls and highlights regional specificity differentiating typical and atypical clinical AD phenotypes. Both control and AD T1w/T2w-ratios appear to be influenced by myelin density as well as iron accumulation and microglia density. In AD, the T1w/T2w-ratio is additionally affected by Aβ and pTau burden and neuro-axonal damage. These findings suggest that the T1w/T2w-ratio may reflect various cortical microstructural changes occurring in AD and its subtypes.

In controls, the observed cortical T1w/T2w-ratio follows a pattern reflective of expected high and low cortical myelin density areas, consistent with previous studies [7, 50, 51]. Namely, a finding of high T1w/T2w-ratio in pre- and postcentral gyri and low T1w/T2w-ratio in inferior frontal and temporal areas. This provides further support for its myelin mapping ability and serves as an anatomical validation of our approach. In AD, the overall cortical T1w/T2w-ratio is decreased, with a more pronounced decrease in the right hemisphere and most prominently in the medial temporal lobe. Previous studies have also reported a decrease in the T1w/T2w-ratio in AD, though these changes were typically more localized as opposed to widespread effects found in the current study, possibly due to an overall earlier disease stage in these in vivo studies [12, 13, 15, 16]. Luo et al. (2019) similarly identified the medial temporal lobe as the region showing the greatest decrease in T1w/T2w-ratio, with a progressive decline in T1w/T2w-ratios across increasingly advanced stages of the disease. In contrast with the findings of the current study, Pelkmans et al. reported an increased cortical T1w/T2w-ratio in AD. Their approach involved unstandardized T1w/T2w-ratio measures in a “probable” AD cohort, confirmed by abnormal Aβ CSF levels. The differences in approach and cohort, with the current study focusing on predominantly pathologically confirmed end-stage AD cases, may explain these contrasting results.

Additionally, when investigating differences between clinical phenotypes, the current study observed lower (para)hippocampal T1w/T2w-ratio in typical AD compared to atypical AD. It is characteristic for atypical AD phenotypes to show a more cortical dominant pathology pattern, with involvement of the hippocampal regions at a more advanced disease stage [25]. Therefore, the difference in (para)hippocampal T1w/T2w-ratio may be attributed to the later involvement of these regions in atypical AD disease progression. This sensitivity of the T1w/T2w-ratio to different disease trajectories has not been explored before but may enhance its utility in distinguishing AD phenotypes.

As well described in the field, aberrant aggregation of Aβ and pTau, as well as neuro-axonal damage is observed in AD cases [52, 53]. In addition, we find evidence for (regional) myelin loss, microglia mediated neuroinflammation and iron accumulation as was also previously observed in AD [2, 52, 54, 55]. When integrating T1w/T2w-ratio with histological data, this study confirms the previous hypothesis that the T1w/T2w-ratio maps myelin, as an increase in T1w/T2w-ratio results in an increase in myelin, in both control and AD cases [7, 9, 23]. In AD the T1w/T2w-ratio also shows to associate with Aβ and pTau aggregation, as well as neuro-axonal damage, which provides evidence that the T1w/T2w-ratio is a marker not only of myelin but of overall cortical integrity.

Considering the lower T1w/T2w-ratio in AD compared to controls, the negative association with pTau is as predicted, but the positive associations with Aβ aggregation and neuro-axonal damage are unexpected. A positive association suggests that, when Aβ and NfL accumulate due to disease progression, T1w/T2w-ratios in AD would approach those of healthy controls, which is contradictory in view of the decreased T1w/T2w-ratio in AD but could be explained by an inflammation-mediated process. These findings are consistent with prior amyloid PET and CSF studies, which similarly report positive associations with T1w/T2w-ratios [16, 17]. Notably, a positive relationship between both the histological markers for Aβ burden and myelin density was observed, which may indicate a compensatory remyelination response, more pronounced in regions of greater amyloid accumulation. This coupling could indicate that the effect of Aβ on T1w/T2w-ratio is partly mediated through myelin [3, 56, 57]. More research into Aβ and myelin remyelination associations is required to confirm this speculation. Recent research indicates both Aβ and NfL are tightly associated with inflammation and the inflammatory cascade, such that an increase in these markers could lead to an elevation in inflammatory mechanisms [58, 59]. Consequently, this could suggest that an Aβ and NfL mediated increase is followed by an indirect inflammation-mediated decrease in T1w/T2w-ratio. This, together with the lowering effect of pTau aggregation, would result in a net decrease of the T1w/T2w-ratio. Furthermore, this study finds evidence for correlations between both Aβ and NfL, and microglia-mediated inflammation, further suggesting an inflammatory link, which requires more research to fully elucidate.

In both controls and AD, we observed an association between T1w/T2w-ratio and iron accumulation, reflecting the T2 sensitivity to ferromagnetic effects [60]. In controls, higher T1w/T2w-ratio corresponded to lower iron accumulation, while in AD patients, higher T1w/T2w-ratio was associated with higher iron levels. Since iron content relates to T2w hypo-intensities, an increase in T1w/T2w-ratio is expected, making the findings in controls counterintuitive. A possible explanation could be differences in iron storage, which tends to accumulate in focal hotspots in AD (e.g., Aβ plaques and microglia) but may remain more evenly regulated in healthy tissue [61–64]. This could lead to divergent effects of iron on the T1w/T2w-ratio or reflect different compensatory mechanisms to maintain tissue homeostasis, such as variations in glial reuptake [64].

General microglia density showed one of the stronger (negative) relationships with T1w/T2w-ratio, particularly in AD, whereas microglia-mediated inflammation (morphological inflammation ratio) showed no associations. The inflammation metric itself displayed minimal variance, particularly in AD, possibly due to postmortem effects driving microglia into an ameboid (inflammatory) state, which was the most frequently observed phenotype. This could obscure true inflammatory differences and make it challenging to find associations with the T1w/T2w-ratio. The strong link with microglia density could simply reflect microglia cell loss but could also suggest a possible inflammatory effect not captured by our morphology analysis, which could be assessed in future research by applying a more extensive set of inflammatory markers (e.g., CD68, GFAP, CRP, IL-6).

Previous studies have reported that the APOE4 genotype may influence the T1w/T2w-ratio, even in a dose-dependent manner, suggesting that carrying more APOE4 alleles is associated with greater reductions in T1w/T2w-ratio measurements [16, 65]. These studies reason that an increased Aβ burden, broader effects of neurodegeneration or even a direct impairment of myelination mediated by APOE4 could be the underlying factors of this observed effect [66–68]. In the current study, however, no effect of APOE4 on T1w/T2w-ratio measurements was observed. This may be due to the late disease stage characteristic of this cohort, given that previous studies have predominantly identified APOE4 effects during prodromal stages of the disease. The extensive pathological burden present in cases of the current study may obscure APOE4-mediated effects that could be more pronounced in earlier disease stages.

An important finding is that the strong link between observed histological markers and the T1w/T2w-ratio when assessing between-region effects within individuals, are not observed when assessing within-region effects (i.e., whole-brain effects that are consistent within regions), or region-by-region effects. This reveals that T1w/T2w-ratio change reliably reflects microstructural variation on a within-subject level, but inter-individual differences in the same region do not consistently map onto histological change. This may be due to the modest sample size of this study, regional heterogeneity, or non-linear effects between microstructural changes and T1w/T2w-ratio that linear models cannot fully capture. Nonetheless, the clear effects found across individual brains suggest the T1w/T2w-ratio can reflect individual microstructural patterns and might be a well-suited tool for personalized assessment, which could be especially useful in disease monitoring. A fundamental challenge in applying the T1w/T2w-ratio in early disease, however, is its demonstrated non-specificity. In normal aging the T1w/T2w-ratio reflects primarily myelin, in AD it reflects multiple microstructural changes, but the transition moment is unclear. This underscores the continued necessity of concurrent AD biomarker confirmation. With current knowledge, the T1w/T2w-ratio would be most effective as a disease monitoring tool in biomarker confirmed AD cases.

Overall, these findings offer a plausible explanation to the ongoing debate in the field regarding the ability of the T1w/T2w-ratio to map myelin or overall tissue integrity. It appears to primarily map myelin in healthy brains, but when pathological processes affect brain tissue microstructure, as seen in AD, the T1w/T2w-ratio also reflects these changes. Previous studies agree with this explanation and hypothesized that the T1w/T2w-ratio is influenced by pathological processes [9, 22, 23], but only few have explored this further. These few studies have confirmed associations between the T1w/T2w-ratio and measures of Aβ and pTau [14, 17], aligning with our findings; yet these studies assessed neuropathology indirectly through global CSF measurements [14, 17], alternative MRI techniques like quantitative susceptibility mapping and diffusion tensor imaging [11, 69], or assessed only few markers [20, 70, 71]. Our study is the first to include a broad set of ground-truth measured histological markers across the cortex, providing unique insights into the T1w/T2w-ratio on a regional level.

Although the T1w/T2w-ratio is not specific to myelin changes in AD, its demonstrated sensitivity to broader pathophysiological changes supports its potential utility in monitoring disease progression across the AD continuum. Onset of Aβ accumulation can precede cognitive symptoms by decades, following a spatiotemporal progression from association cortices to medial temporal regions, where pTau pathophysiology is initiated and drives neuro-axonal deterioration throughout the brain [1, 30, 32, 72]. Myelin integrity changes similarly exhibit a clear spatiotemporal pattern across both development and degeneration, as demonstrated using the T1w/T2w-ratio by Grydeland et al. [73], and have been shown to be affected by AD pathophysiology as well as potentially preceding it [2, 3]. The findings of this study suggest that the T1w/T2w-ratio warrants further investigation for its spatiotemporal sensitivity to these pathophysiological changes along the AD continuum, with the aim of capturing early microstructural changes and monitoring them throughout disease progression. While prior studies have examined the T1w/T2w-ratio across disease stage [12], future research would benefit from studying more incremental steps in the temporal progression of AD in combination with well-characterized pathophysiological markers, such as CSF Aβ and pTau biomarkers.

The current study has several limitations. First, the postmortem nature indicates that all AD subjects were in late-stage disease by exhibiting an advanced pathological burden, and may have been affected by postmortem effects, such as tissue decomposition or change in fluid composition [74]. However, the concordance with previous observations and strong postmortem-to‐antemortem correlation of the T1w/T2w-ratio indicate a robust translational potential. Another limitation of the current study is the risk of partial volume effects, arising from the 3 mm slice thickness of the T2-weighted acquisition combined with the increased cortical atrophy expected in aging and AD. Partial volume effects were limited through a strict signal extraction approach which was shown to be robust in the sensitivity analysis. However, partial volume effects cannot fully be avoided, and results should be interpreted with this caveat in mind. Additionally, as this study focuses on cortical grey matter, the sensitivity of the T1w/T2w-ratio to pathological processes in white matter structures remains unclear. While the current study advances the understanding of the T1w/T2w-ratio in mapping microstructural changes, larger sample sizes could provide better sensitivity to capture regional effects and longitudinal data across disease stages would allow for assessing the true ability to monitor microstructural change in AD. Additionally, a more extensive investigation into inflammation markers or cytoarchitectural changes such as seen in cell loss, would fill some of the gaps still left by the current study to better characterize the T1w/T2w-ratio reflection of microstructural changes.

## Conclusions

The findings of this paper provide evidence that the T1w/T2w-ratio in AD is not exclusively sensitive to myelin, but more broadly reflects microstructural changes affecting tissue integrity, which becomes especially relevant under the pathophysiological conditions of AD. In biomarker confirmed AD cases, T1w/T2w-ratio is a potentially viable tool for longitudinal, within-subject monitoring of disease-related neuropathological progression beyond myelin.

## Supplementary Information

Supplementary Material 1.

Supplementary Material 2.

## Data Availability

Data from the current study are available upon reasonable request.

## Abbreviations

3T: 3 tesla
Aβ: amyloid-beta
AD: Alzheimer’s disease
APOE4: apolipoprotein E ε4 allele
AtypAD: atypical Alzheimer’s disease
aSyn: alpha-synuclein
BNE: brain net Europe
CAA: cerebral amyloid angiopathy
CSF: cerebrospinal fluid
DAB: 3,3’-diaminobenzidine
FDR: false discovery rate
GM: grey matter
GFM: middle frontal gyrus
GPS: superior parietal gyrus
GTM: middle temporal gyrus
HIER: heat-induced epitope retrieval
Hip: hippocampus
IBA1: ionized calcium-binding adapter molecule 1
ICV: intracranial volume
IHC: immunohistochemistry
LATE-NC: limbic-predominant age-related TDP-43 encephalopathy neuropathologic changes
MS: multiple sclerosis
NABCA: normal aging brain collection Amsterdam
NBB: Netherlands brain bank
NFL: neurofilament light chain (antibody)
NfL: neurofilament light chain
NFT: neurofibrillary tangle
OC: occipital cortex
PCC: posterior cingulate gyrus
PFA: paraformaldehyde
PLP: proteolipid protein
PMD: postmortem delay
Precun: precuneus
pTau: phosphorylated tau
T1w/T2w-ratio: ratio of T1-weighted to T2-weighted MRI signal intensity
TBS: tris-buffered saline
TDP-43: TAR DNA-binding protein 43
TE: echo time
TR: repetition time
Tris–HCl: tris(hydroxymethyl)aminomethane hydrochloride buffer
TypAD: typical Alzheimer’s disease
WM: white matter
