# Supplementary material for: T1-weighted/T2-weighted ratio reflects microstructural changes in Alzheimer’s disease
Source: Alzheimers Res Ther. 2026 May 27;18:175. doi: 10.1186/s13195-026-02093-6 (PMC13404876; doi:10.1186/s13195-026-02093-6)
Supplement: Supplementary file 1 — Supplementary Material 1. [file 13195_2026_2093_MOESM1_ESM.docx]

**Table 1. Overview individual cases**

| **Case code** | **Subtype** | **Histology available** | **Sex** | **Age at death** | **Cause of death** | **PMD** | **Disease duration** | **CDR** | **APOE** | **Thal** | **Braak NFT** | **Braak**  **asyn** | **LATE** | **CAA-Type** | **GCA** | **MTA** | **PCA** | **Fazekas** | **NWBV** | **NGMV** | | **NWMV** | |
| --- | --- | --- | --- | --- | --- | --- | --- | --- | --- | --- | --- | --- | --- | --- | --- | --- | --- | --- | --- | --- | --- | --- | --- |
| **Typical AD** | | | | | | | | | | | | | | | | | | | | | | |  |
| AD-02 | Typical | Yes | M | 60 | Euthanasia | 8:35 | 2 | NA | 3/3 | 5 | 6 | 0 | 0 | 1 | 0 | 0 | 1 | 3 | 69.20 | 37.62 | | 31.58 | |
| AD-04 | Typical | Yes | M | 68 | Euthanasia | 9:15 | 6 | NA | 3/3 | 5 | 5 | 0 | 0 | 2 | 2 | 1 | 1 | 1 | 69.83 | 39.61 | | 30.22 | |
| AD-05 | Typical | Yes | M | 69 | Pulmonary infection | 11:55 | 11 | 3 | 3/4 | 5 | 5 | 0 | 0 | 1 | 1 | 3 | 2 | 0 | 57.27 | 33.81 | | 23.46 | |
| AD-09 | Typical | No | M | 84 | Euthanasia | 5:53 | 13 | 1 | 3/4 | 5 | 4 | 0 | 0 | 1 | 1 | 3 | 2 | 1 | 64.22 | 32.92 | | 31.30 | |
| AD-13 | Typical | Yes | F | 80 | Euthanasia | 7:05 | 7 | 1 | 3/3 | 5 | 4 | 0 | 1 | 1 | 1 | 2 | 1 | 1 | 65.59 | 37.87 | | 27.72 | |
| AD-17 | Typical | Yes | M | 53 | Palliative sedation | 9:00 | 5 | 3 | 3/3 | 5 | 6 | 0 | 0 | 1 | 3 | 1 | 2 | 0 | 56.38 | 30.51 | | 25.88 | |
| AD-18 | Typical | Yes | M | 64 | Cachexia | 7:55 | 12 | 3 | 3/4 | 5 | 6 | Amyg | 0 | 2 | 2 | 4 | 1 | 0 | 54.48 | 30.90 | | 23.59 | |
| AD-21 | Typical | Yes | M | 84 | Euthanasia | 8:35 | 14 | NA | 3/3 | 3 | 4 | 0 | 0 | 0 | 2 | 2 | 1 | 2 | 58.18 | 35.52 | | 22.66 | |
| AD-22 | Typical | Yes | M | 77 | Drug overdoses | 9:05 | 10 | NA | 4/4 | 5 | 6 | 0 | 1 | 1 | 1 | 1 | 1 | 3 | 62.23 | 34.82 | | 27.41 | |
| AD-23 | Typical | Yes | M | 65 | Euthanasia | 9:20 | 7 | 1 | 3/4 | 5 | 5 | 0 | 0 | 1 | 0 | 1 | 1 | 1 | 69.69 | 41.80 | | 27.88 | |
| AD-25 | Typical | Yes | M | 63 | Pulmonary embolism | 8:45 | 10 | 2 | 3/4 | 5 | 6 | 0 | 0 | 1 | 1 | 1 | 3 | 0 | 60.70 | 35.36 | | 25.34 | |
| AD-30 | Typical | Yes | F | 61 | End-Stage AD | 7:40 | 6 | NA | 4/4 | 5 | 6 | 0 | 2 | 1 | 1 | 3 | 3 | 0 | 56.24 | 33.96 | | 22.28 | |
| AD-31 | Typical | Yes | F | 53 | End-Stage AD | 6:30 | 7 | 2 | 2/3 | 5 | 6 | 0 | 0 | 1 | 1 | 2 | 1 | 3 | 61.71 | 36.52 | | 25.18 | |
| AD-32 | Typical | Yes | M | 60 | Euthanasia | 7:05 | 2 | 1 | 4/4 | 5 | 6 | 0 | 0 | 1 | 1 | 2 | 2 | 1 | 65.42 | 37.90 | | 27.52 | |
| AD-35 | Typical | Yes | M | 67 | Euthanasia | 8:00 | 10 | 1 | 3/4 | 5 | 6 | Amyg | 0 | 1 | 1 | 3 | 2 | 0 | 57.06 | 35.04 | | 22.02 | |
| AD-37 | Typical | Yes | M | 79 | Unknown | 8:05 | 8 | NA | 3/3 | 5 | 5 | 2 | 3 | 1 | 2 | 4 | 1 | 3 | 51.49 | 38.19 | | 13.29 | |
| AD-38 | Typical | Yes | F | 89 | End-Stage AD | 6:35 | 8 | 3 | 4/4 | 5 | 5 | Amyg | 2 | 1 | 1 | 4 | 2 | 3 | 62.35 | 38.04 | | 24.31 | |
| AD-40 | Typical | Yes | M | 87 | Pneumonia | 6:40 | 10 | NA | 3/4 | 5 | 6 | 0 | 2 | 1 | 3 | 4 | NA | 3 | 46.23 | 23.56 | | 22.67 | |
| AD-44 | Typical | No | M | 78 | Euthanasia | 9:40 | 2 | 0,5 | 4/4 | 5 | 4 | 0 | 0 | 1 | 2 | 2 | NA | 2 | 66.61 | 37.41 | | 29.20 | |
| AD-46 | Typical | No | M | 77 | Euthanasia | 5:00 | 2 | NA | NA | 5 | 5 | 0 | 0 | 1 | NA | 2 | NA | 0 | 62.83 | 35.54 | | 27.29 | |
| AD-48 | Typical | No | M | 64 | Pneumonia | 8:20 | 8 | NA | NA | 5 | 5 | 0 | 0 | 1 | NA | 2 | NA | 0 | 52.87 | 44.81 | | 8.05 | |
| AD-51 | Typical | No | M | 56 | Jawbone infection | 8:45 | 2 | NA | 3/4 | 5 | 5 | 0 | 0 | 1 | NA | 2 | NA | 3 | 66.66 | 36.72 | | 29.94 | |
| AD-52 | Typical | No | F | 70 | Euthanasia | 7:00 | 1 | NA | NA | 5 | 5 | 0 | 0 | 2 | NA | 1 | 2 | 1 | 65.00 | 36.86 | | 28.14 | |
| AD-55 | Typical | No | F | 71 | End-Stage AD | 8:00 | NA | NA | NA | 3 | 6 | 1 | 1 | 1 | 4 | 4 | 3 | 3 | 54.75 | 23.58 | | 31.17 | |
| AD-57 | Typical | No | M | 82 | Infection | 7:55 | 12 | NA | NA | 5 | 6 | 0 | 1 | 1 | NA | 4 | NA | 2 | 55.42 | 31.04 | | 24.38 | |
| **Atypical AD** | | | | | | | | | | | | | | | | | | | | | | |  |
| AD-16 | behavioural | Yes | M | 77 | Euthanasia | 7:00 | 4 | 1 | 3/4 | 5 | 4 | 0 | 0 | 2 | 0 | 0 | 0 | 1 | 72.98 | 40.75 | | 32.23 | |
| AD-20 | behavioural | Yes | M | 73 | Cachexia | 7:20 | 10 | 3 | 3/4 | 5 | 5 | 5 | 0 | 1 | 3 | 3 | 3 | 2 | 51.65 | 24.97 | | 26.68 | |
| AD-27 | behavioural | Yes | F | 73 | Pneumothorax | 6:15 | 1 | 3 | 3/4 | 5 | 6 | 0 | 1 | 2 | 0 | 3 | 1 | 0 | 59.90 | 37.22 | | 22.68 | |
| AD-07 | dysexecutive | Yes | M | 59 | Euthanasia | 6:30 | 2 | 2 | 4/4 | 5 | 5 | 5 | 2 | 1 | 0 | 1 | 2 | 2 | 74.42 | 42.05 | | 32.37 | |
| AD-08 | dysexecutive | Yes | F | 78 | Cachexia | 7:30 | 4 | 3 | 3/4 | 5 | 5 | 3 | 0 | 1 | 1 | 3 | 2 | 3 | 60.43 | 33.03 | | 27.39 | |
| AD-11 | dysexecutive | Yes | M | 37 | Euthanasia | 11:11 | 5 | 1 | 2/3 | 5 | 6 | 0 | 0 | 1 | 0 | 1 | 1 | 0 | 68.30 | 38.31 | | 30.00 | |
| AD-12 | dysexecutive | Yes | M | 58 | Cachexia | 8:55 | 2 | 3 | 3/4 | 5 | 6 | 0 | 0 | 1 | 1 | 1 | 2 | 0 | 66.77 | 37.82 | | 28.95 | |
| AD-19 | dysexecutive | Yes | M | 59 | Dysphagia | 5:35 | 3 | 3 | 3/4 | 5 | 5 | 0 | 0 | 1 | 1 | 3 | 2 | 2 | 55.45 | 34.39 | | 21.06 | |
| AD-26 | logopenic | Yes | F | 72 | End-Stage AD | 5:05 | 5 | 1 | 2/3 | 5 | 6 | 0 | 0 | 2 | 2 | 2 | 2 | 2 | 53.30 | 26.85 | | 26.45 | |
| AD-34 | logopenic | Yes | M | 75 | End-Stage AD | 9:45 | 5 | 3 | 4/4 | 5 | 6 | 0 | 3 | 1 | 3 | 4 | 2 | 3 | 55.60 | 30.94 | | 24.66 | |
| AD-39 | logopenic | Yes | F | 67 | End-Stage AD | 8:15 | 1 | 3 | 3/3 | 5 | 5 | 0 | 0 | 1 | 1 | 2 | 2 | 1 | 57.98 | 32.14 | | 25.84 | |
| AD-54 | logopenic | No | F | 71 | Euthanasia | 8:30 | 10 | NA | NA | 5 | 6 | 0 | 0 | 2 | NA | 3 | 3 | 0 | 58.38 | 30.01 | | 28.36 | |
| AD-10 | visuospatial | Yes | M | 62 | Palliative sedation | 8:15 | 5 | 3 | 3/4 | 4 | 6 | 0 | 0 | 1 | 2 | 2 | 3 | 3 | 52.40 | 28.25 | | 24.14 | |
| AD-15 | visuospatial | Yes | M | 67 | Cachexia | 6:35 | 9 | 3 | 3/4 | 5 | 6 | 0 | 0 | 2 | 3 | 2 | 2 | 0 | 58.00 | 33.13 | | 24.87 | |
| AD-28 | visuospatial | Yes | F | 60 | Euthanasia | 10:50 | 2 | 2 | 3/3 | 5 | 6 | 0 | 0 | 2 | 1 | 1 | 3 | 1 | 65.28 | 38.02 | | 27.26 | |
| AD-29 | visuospatial | Yes | M | 68 | End-Stage AD | 6:22 | 3 | NA | 3/4 | 5 | 6 | 0 | 0 | 2 | 1 | 3 | 1 | 2 | 56.26 | 38.29 | | 17.97 | |
| AD-41 | visuospatial | Yes | M | 56 | Euthanasia | 9:00 | 2 | NA | 3/4 | 5 | 5 | 4 | 0 | 2 | NA | 0 | 2 | 1 | 62.14 | 33.91 | | 28.24 | |
| AD-45 | visuospatial | No | F | 65 | End-Stage AD | 7:20 | 7 | NA | NA | 5 | 6 | 0 | 0 | 2 | 3 | 4 | 3 | 0 | 48.83 | 26.64 | | 22.20 | |
| AD-56 | visuospatial | No | F | 54 | Euthanasia | 10:30 | 1 | NA | NA | 5 | 6 | Amyg | 2 | 2 | NA | 1 | NA | 0 | 67.65 | 38.55 | | 29.10 | |
| AD-06 | Atypical | Yes | M | 65 | Cardiac arrest | 7:50 | 7 | NA | 3/3 | 4 | 5 | 0 | 0 | 1 | 0 | 1 | 1 | 2 | 70.93 | 38.48 | | 32.45 | |
| **AD clinical phenotype TBD** | | | | | | | | | | | | | | | | | | | | |  |  |  |
| AD-36 | NA | Yes | M | 70 | End-Stage AD | 5:50 | 12 | NA | 4/4 | 5 | 6 | 0 | 2 | 1 | 2 | 4 | 1 | 3 | 58.03 | 31.63 | | 26.40 | |
| AD-42 | NA | Yes | F | 75 | Euthanasia | 4:20 | 3 | NA | 3/4 | 5 | 5 | 0 | 0 | 2 | NA | 1 | NA | 2 | 67.27 | 37.93 | | 29.33 | |
| AD-43 | NA | No | F | 85 | End-Stage AD | 7:40 | 4 | NA | NA | 5 | 5 | 0 | 0 | 1 | NA | 4 | NA | 3 | 61.99 | 29.16 | | 32.83 | |
| AD-49 | NA | No | M | 76 | End-Stage AD | 8:30 | 10 | NA | NA | 5 | 6 | 0 | 2 | 1 | NA | 3 | NA | 0 | 58.42 | 35.55 | | 22.87 | |
| AD-53 | NA | No | M | 65 | End-Stage AD | 5:30 | NA | NA | NA | 5 | 6 | 6 | 0 | 2 | NA | 4 | NA | 1 | 55.12 | 32.10 | | 23.03 | |
| AD-59 | NA | No | F | 68 | Pneumonia | 5:38 | NA | NA | NA | 5 | 6 | 0 | 2 | 2 | NA | 2 | 1 | 3 | 59.96 | 34.47 | | 25.49 | |
| **Control** | | | | | | | | | | | | | | | | | | | | | | | |
| Control 1 | Control | Yes | M | 68 | Euthanasia | 8:40 | NA | NA | 3/4 | 2 | 1 | 0 | 0 | 2 | 0 | 0 | 0 | 0 | 75.81 | 45.63 | | 30.18 | |
| Control 2 | Control | No | F | 81 | Euthanasia | 8:15 | NA | NA | 3/4 | 3 | 2 | 0 | 0 | 1 | NA | 1 | NA | 2 | 65.11 | 36.48 | | 28.63 | |
| Control 3 | Control | No | M | 82 | Euthanasia | 8:15 | NA | NA | 3/3 | 1 | 2 | 3 | 0 | 0 | NA | 1 | NA | 1 | 65.58 | 37.92 | | 27.66 | |
| Control 4 | Control | No | F | 57 | Euthanasia | 9:50 | NA | NA | 3/4 | 1 | 0 | 0 | 0 | 0 | NA | 0 | NA | 0 | 76.60 | 47.93 | | 28.66 | |
| Control 5 | Control | Yes | F | 63 | Euthanasia | 8:00 | NA | NA | 2/3 | 0 | 0 | 0 | 0 | 0 | 0 | 0 | 0 | 0 | 76.65 | 42.18 | | 34.47 | |
| Control 6 | Control | No | M | 57 | Euthanasia | 10:0 | NA | NA | 3/3 | 1 | 1 | 0 | 0 | 0 | NA | 0 | NA | 0 | 78.69 | 42.63 | | 36.06 | |
| Control 7 | Control | No | M | 76 | Euthanasia | 6:30 | NA | NA | 3/4 | 0 | 1 | 0 | 0 | 2 | NA | 2 | NA | 1 | 62.80 | 35.98 | | 26.81 | |
| Control 8 | Control | Yes | M | 82 | Palliative sedation | 10:30 | NA | NA | 2/3 | 1 | 1 | 0 | 0 | 0 | 0 | 0 | 1 | 2 | 68.83 | 38.20 | | 30.64 | |
| Control 9 | Control | Yes | M | 85 | Euthanasia | 9:20 | NA | NA | 3/3 | 1 | 1 | 0 | 0 | 0 | 0 | 1 | 0 | 2 | 66.50 | 36.09 | | 30.41 | |
| Control 10 | Control | No | M | 74 | GI carcinoma | 9:20 | NA | NA | 3/3 | 1 | 1 | 0 | 0 | 2 | NA | 1 | NA | 0 | 72.44 | 40.89 | | 31.55 | |
| Control 11 | Control | Yes | M | 67 | Euthanasia | 8:25 | NA | NA | 3/4 | 1 | 2 | 0 | 0 | 0 | 0 | 0 | 0 | 1 | 67.85 | 37.01 | | 30.84 | |
| Control 12 | Control | Yes | F | 76 | Euthanasia | 7:30 | NA | NA | 3/3 | 2 | 1 | 0 | 0 | 0 | 0 | 1 | 0 | 2 | 68.74 | 41.56 | | 27.18 | |
| Control 13 | Control | Yes | M | 67 | Liver failure | 8:35 | NA | NA | 2/3 | 1 | 1 | 0 | 0 | 2 | 0 | 1 | 0 | 0 | 71.68 | 42.02 | | 29.66 | |
| Control 14 | Control | No | F | 76 | Euthanasia | 9:0 | NA | NA | NA | 1 | 2 | 0 | 0 | 2 | NA | 2 | NA | 0 | 67.12 | 40.77 | | 26.35 | |
| Control 15 | Control | No | F | 87 | Urinary tract infection | 8:30 | NA | NA | 3/3 | 0 | 1 | 0 | 0 | 0 | NA | 1 | NA | 0 | 68.52 | 43.70 | | 24.82 | |
| Control 16 | Control | Yes | F | 72 | Cardiac arrest | 8:35 | NA | NA | 3/3 | 0 | 0 | 0 | 0 | 0 | 0 | 0 | 0 | 1 | 74.60 | 43.49 | | 31.12 | |
| Control 17 | Control | Yes | F | 69 | Pulmonary embolism | 7:20 | NA | NA | 3/3 | 1 | 1 | 1 | 0 | 0 | 0 | 1 | 0 | 2 | 71.20 | 41.04 | | 30.16 | |
| Control 18 | Control | Yes | M | 59 | Euthanasia | 8:0 | NA | NA | 3/4 | 2 | 1 | 0 | 0 | 0 | 0 | 1 | 1 | 0 | 71.61 | 40.37 | | 31.24 | |
| Control 19 | Control | No | M | 74 | Cancer | 16:0 | NA | NA | NA | 2 | 1 | 0 | 0 | 0 | NA | 2 | NA | 0 | 65.31 | 39.31 | | 26.00 | |
| Control 20 | Control | No | M | 72 | Oesophagus cancer | 11:45 | NA | NA | 2/3 | 3 | 2 | 3 | 0 | 2 | NA | 1 | NA | 0 | 68.31 | 38.60 | | 29.71 | |
| Control 21 | Control | Yes | M | 77 | Pneumonia | 11:45 | NA | NA | 2/3 | 1 | 1 | 0 | 0 | 0 | 1 | 2 | 2 | 0 | 66.57 | 35.17 | | 31.39 | |
| Control 22 | Control | Yes | F | 79 | Unknown | 6:15 | NA | NA | NA | 3 | 2 | 0 | 0 | 0 | 0 | 0 | 0 | 0 | 75.36 | 44.01 | | 31.34 | |
| Control 23 | Control | Yes | F | 78 | Unknown | 5:25 | NA | NA | 3/3 | 1 | 1 | 1 | 0 | 0 | 0 | 0 | 0 | 2 | 69.19 | 38.10 | | 31.09 | |
| Control 24 | Control | No | F | 77 | GI carcinoma | 4:30 | NA | NA | 3/3 | 2 | 2 | 2 | 0 | 0 | NA | 1 | NA | 2 | 72.76 | 43.28 | | 29.49 | |
| Control 25 | Control | Yes | F | 59 | Euthanasia | 4:30 | NA | NA | 3/3 | 0 | 0 | 0 | 0 | 0 | 0 | 0 | 1 | 2 | 70.22 | 41.72 | | 28.50 | |
| Control 26 | Control | Yes | F | 71 | Lung carcinoma | 8:10 | NA | NA | 3/4 | 2 | 1 | 0 | 0 | 1 | 1 | 0 | 1 | 2 | 70.37 | 40.06 | | 30.31 | |
| Control 27 | Control | Yes | M | 74 | Euthanasia | 10:20 | NA | NA | 3/3 | 3 | 2 | 0 | 0 | 0 | 1 | 1 | 1 | 1 | 62.42 | 37.44 | | 24.98 | |
| PMD is presented in hours:minutes, age and disease duration are presented in years and NWBV, NGMV, and NWMV in percentage of estimated intracranial volume. APOE describes genotyping of present APOE isoforms.  amyg = amygdala predominant, CAA = cerebral amyloid angiopathy, CDR = clinical dementia score, F = female, GCA = global cortical atrophy, GI = gastrointestinal, asyn = alpha-synucleinopathy, LATE = Limbic-predominant age-related TDP-34 encephalopathy, M = male, MTA = medial temporal lobe atrophy, NA = not available, NFT = neurofibrillary tangles, NGMV = normalized grey matter volume, NWMV = normalized white matter volume, NWBV = normalized whole brain volume, PCA = parietal cortical atrophy, PMD = postmortem delay. | | | | | | | | | | | | | | | | | | | | | | | |

**Supplementary Table 2. Ante-mortem MRI scan specifications**

| Case | Months before death | Scanner | Sequence  T1w&T2w | Dimensions | In/Through-plane resolution | TE | TR | Flip angle |  |
| --- | --- | --- | --- | --- | --- | --- | --- | --- | --- |
| AD-6 | 17 | Philips Ingenuity | TFE | 194x256x256 | 1/1 | 4.5 | 7.9 | 8 |  |
|  |  |  | TSE | 46x512x512 | 3/3.3 | 19 | 3867 | 90 |  |
| AD-7 | 5 | Siemens Avanto | MPRAGE | 176x256x256 | 1/1 | 2.9 | 1900 | 15 |  |
|  |  |  | TSE | 40x512x408 | 3/3.9 | 96 | 4000 | 150 |  |
| AD-11 | 1 | GE Discovery MR750 | FSPGR | 176x256x256 | 1 | 2.9 | 6.7 | 15 |  |
|  |  |  | TSE | 47x512x512 | 3/3 | 114.8 | 4246 | 111 |  |
| AD-13 | 10 | Philips Ingenuity | TFE | 194x256x256 | 1/1 | 4.5 | 7.9 | 8 |  |
|  |  |  | TSE | 46x512x512 | 3/3.3 | 19 | 3867 | 90 |  |
| AD-16 | 5 | Philips Ingenuity | TFE | 194x256x256 | 1/1 | 4.5 | 7.9 | 8 |  |
|  |  |  | TSE | 46x512x512 | 3/3.3 | 19 | 3867 | 90 |  |
| AD-23 | 22 | Philips Ingenuity | TFE | 194x256x256 | 1/1 | 4.5 | 7.9 | 8 |  |
|  |  |  | TSE | 46x512x512 | 3/3.3 | 19 | 3867 | 90 |  |
| AD-39 | 16 | Siemens Magnetom Vida | MPRAGE | 192x256x256 | 0.9/0.9 | 2.3 | 2300 | 8 |  |
|  |  |  | TSE | 39x512x512 | 3/3.9 | 74 | 5700 | 150 |  |
| AD-46 | 17 | Siemens Magnetom Vida | MPRAGE | 192x256x256 | 0.9/0.9 | 2.3 | 2300 | 8 |  |
|  |  |  | TSE | 39x512x512 | 3/3.9 | 74 | 5700 | 150 |  |
| AD-52 | 15 | Siemens Magnetom Vida | MPRAGE | 192x256x256 | 0.9/0.9 | 2.3 | 2300 | 8 |  |
|  |  |  | TSE | 39x512x512 | 3/3.9 | 74 | 5700 | 150 |  |
| Case numbers match numbers in supplementary table 1. All cases are diagnosed AD cases. TE = echo time, TR = repetition time. | | | | | | | | | |

**Supplementary Table 3. Cohort characteristics histology subset**

|  | **Control** | **AD** | **Typical AD** | **Atypical AD** |
| --- | --- | --- | --- | --- |
|  | n= 16 | n= 36 | n= 17 | n= 17 |
| **Sex**  (% Female) | 8  (50.0%) | 10  (27.8%) | 4  (23.6%) | 5  (29.4%) |
| **Age at death**  (In years) | 72 (±8)  [59 – 85] | 68 (±11)  [37 – 89] | 69 (±11)  [53 – 89] | 65 (±10)  [37 – 78] |
| **Age at Onset**  (In years) | NA | 63 (±10)  [32 – 81] | 61 (±10)  [46 – 81] | 61 (±10)  [32 – 74] |
| **Disease duration**  (In years) | NA | 6 (±4)  [1 - 14] | 8 (±4)  [2 - 14] | 4 (±3) ^###^  [1 - 10] |
| **Clinical Subtyping**  Typical/Atypical | NA | 17/17 NA = 2 | NA | NA |
| **APOE genotyping**  APOE4 carrier | 4 (26.7%)  NA = 1 | 24 (66.6%)***** | 10 (58.8%) | 12 (70.6%) |
| **Postmortem delay**  (Hours:minutes) | 8:33 (±1:51) | 8:10 (±1:40) | 8:34 (±1:21) | 8:07 (±1:44) |
| **MRI** |  |  |  |  |
| **Normalized Whole Brain Volume**  (% ICV) | 70.5 (±3.8) | 60.9 (±6.8)******* | 60.2 (±6.6) | 61.3 (±7.4) |
| **Normalized Grey Matter Volume**  (% ICV) | 40.3 (±3.0) | 35.0 (±4.4)******* | 35.4 (±4.2) | 34.6 (±4.9) |
| **Normalized White Matter Volume**  (% ICV) | 30.2 (±2.1) | 25.9 (±4.0)******* | 24.9 (±4.1) | 26.7 (±4.0) |
| **Global Cortical Atrophy**  0/1/2/3/4 | 13/3/0/0/0 | 7/15/7/5/0*******  NA = 2 | 2/9/4/2/0 | 5/6/2/3/0 NA = 1 |
| **Parietal Cortical Atrophy**  0/1/2/3 | 10/5/1/0 | 1/14/14/5******* NA = 2 | 0/9/5/2 NA = 1 | 1/4/9/3 |
| **Medial Temporal Lobe Atrophy**  0/1/2/3/4 | 9/6/1/0/0 | 3/11/8/8/6******* | 1/5/4/3/4 | 2/6/4/6/2 |
| **Fazekas**  0/1/2/3 | 6/3/7/0 | 10/8/8/10 | 6/4/1/6 | 4/4/6/3 |
| **Pathology** |  |  |  |  |
| **Braak NFT stage**  0/1/2/3/4/5/6 | 3/10/3/0/0/0/0 | 0/0/0/0/3/13/20 ******* | 0/0/0/0/2/5/10 | 0/0/0/0/1/7/9 |
| **Thal Aβ phase**  0/1/2/3/4/5 | 7/4/2/0/0/0 | 0/0/0/1/2/33 ******* | 0/0/0/1/0/16 | 0/0/0/0/2/15 |
| **Braak asyn stage**  0/1/2/3/4/5/6  Amygdala predom | 14/2/0/0/0/0  0 | 27/0/1/1/1/2/0  3 | 13/0/1/0/0/0/0  3 | 13/0/0/1/1/2/0  0 |
| **LATE stage**  0/1/2/3 | 16/0/0/0 | 26/3/5/2 | 11/2/3/1 | 14/1/1/1 |
| **CAA-Type**  0/1/2 | 13/1/2 | 1/25/10 ******* | 1/14/2 | 0/10/7 |
| Data is noted as mean (±standard deviation) [range] or count (ratio %). Significance between control and AD groups is denoted with * = *p* ≤ 0.05, ** = *p* ≤ 0.01, *** = *p* ≤ 0.001. Significance between typical AD and atypical AD groups is denoted with # = *p* ≤ 0.05, ## = *p* ≤ 0.01, ### = *p* ≤ 0.001. NA = not available, ICV = Intracranial Volume, NFT = Neurofibrillary Tangle, Aβ = Amyloid-beta, asyn = alpha-synucleinopathy, LATE = Limbic-predominant Age-related TDP-43 Encephalopathy, CAA = Cerebral Amyloid Angiopathy. | | | | |

**Supplementary Table 4. Significant group differences of histological markers**

|  | **Contrast** | **Region** | **Estimate** | **EMM group1** | **EMM group2** | **SE** | **t ratio** | **p value** | **p adjusted** | **Percent difference** |  |
| --- | --- | --- | --- | --- | --- | --- | --- | --- | --- | --- | --- |
| **Aβ** | Control - Typical AD | Regions Combined | -1.724 | 1.335 | 3.0599 | 0.647 | 2.667 | 0.028 | 0.070 | 78.47 |  |
|  |  | GFM | -2.306 | 1.804 | 4.1096 | 0.772 | 2.985 | 0.010 | **0.030** | 77.98 |  |
|  |  | GPS | -2.596 | 1.077 | 3.6727 | 0.772 | 3.361 | 0.003 | **0.016** | 109.33 |  |
|  |  | PCC | -2.451 | 1.264 | 3.7151 | 0.772 | 3.174 | 0.006 | **0.021** | 98.48 |  |
|  | Control - Atypical AD | Regions Combined | -2.494 | 1.3354 | 3.829 | 0.659 | -3.787 | 0.001 | **0.010** | 96.58 |  |
|  |  | ParaHip | -2.300 | 1.2584 | 3.558 | 0.783 | -2.939 | 0.012 | **0.032** | 95.50 |  |
|  |  | Fusiform | -2.521 | 1.6430 | 4.164 | 0.803 | -3.140 | 0.006 | **0.021** | 86.83 |  |
|  |  | GFM | -3.160 | 1.8038 | 4.964 | 0.783 | -4.038 | 0.000 | **0.005** | 93.38 |  |
|  |  | GTM | -2.778 | 1.4538 | 4.231 | 0.783 | -3.549 | 0.002 | **0.011** | 97.71 |  |
|  |  | GPS | -3.280 | 1.0765 | 4.356 | 0.783 | -4.191 | 0.000 | **0.005** | 120.74 |  |
|  |  | Precun | -2.578 | 1.9717 | 4.549 | 0.786 | -3.278 | 0.004 | **0.018** | 79.06 |  |
|  |  | PCC | -3.024 | 1.2636 | 4.288 | 0.783 | -3.864 | 0.001 | **0.006** | 108.95 |  |
|  |  | OC | -2.005 | 0.7570 | 2.762 | 0.779 | -2.573 | 0.031 | 0.072 | 113.94 |  |
| **pTau** | Typical – Atypical | Precun | -15.662 | 28.347 | 44.009 | 5.409 | -2.895 | 0.012 | **0.019** | 43.9 |  |
| **NfL** | Control - Typical AD | Regions Combined | -3.122 | 7.621 | 10.7432 | 0.799 | 3.906 | 0.001 | **0.004** | 34.00 |  |
|  |  | ParaHip | -3.407 | 6.211 | 9.6184 | 1.225 | 2.782 | 0.016 | **0.033** | 43.04 |  |
|  |  | Fusiform | -3.687 | 5.889 | 9.5765 | 1.293 | 2.851 | 0.013 | **0.028** | 47.68 |  |
|  |  | GFM | -3.170 | 6.760 | 9.9300 | 1.211 | 2.617 | 0.026 | **0.048** | 37.98 |  |
|  |  | GTM | -3.553 | 6.714 | 10.2678 | 1.224 | 2.902 | 0.011 | **0.028** | 41.85 |  |
|  |  | GPS | -3.655 | 7.479 | 11.1338 | 1.211 | 3.018 | 0.008 | **0.024** | 39.28 |  |
|  |  | Precun | -2.901 | 9.123 | 12.0247 | 1.211 | 2.396 | 0.046 | 0.074 | 27.44 |  |
|  |  | OC | -3.487 | 8.078 | 11.5646 | 1.201 | 2.904 | 0.011 | **0.028** | 35.50 |  |
|  | Control - Atypical AD | Regions Combined | -4.685 | 7.6214 | 12.306 | 0.808 | -5.796 | 0.000 | **0.000** | 47.02 |  |
|  |  | Hip | -2.970 | 9.2430 | 12.213 | 1.217 | -2.441 | 0.041 | 0.073 | 27.69 |  |
|  |  | ParaHip | -3.545 | 6.2114 | 9.757 | 1.231 | -2.880 | 0.012 | **0.028** | 44.41 |  |
|  |  | Fusiform | -5.972 | 5.8893 | 11.861 | 1.298 | -4.602 | 0.000 | **0.000** | 67.29 |  |
|  |  | GFM | -4.805 | 6.7604 | 11.566 | 1.217 | -3.948 | 0.000 | **0.002** | 52.44 |  |
|  |  | GTM | -5.122 | 6.7144 | 11.836 | 1.230 | -4.163 | 0.000 | **0.001** | 55.22 |  |
|  |  | GPS | -6.523 | 7.4786 | 14.002 | 1.217 | -5.360 | 0.000 | **0.000** | 60.74 |  |
|  |  | Precun | -5.235 | 9.1234 | 14.358 | 1.217 | -4.301 | 0.000 | **0.001** | 44.59 |  |
|  |  | PCC | -3.746 | 8.5089 | 12.254 | 1.232 | -3.041 | 0.008 | **0.024** | 36.08 |  |
|  |  | OC | -4.740 | 8.0778 | 12.818 | 1.207 | -3.927 | 0.000 | **0.002** | 45.37 |  |
|  | Typical AD - Atypical AD | GPS | -2.868 | 11.1338 | 14.002 | 1.200 | -2.390 | 0.047 | 0.074 | 22.82 |  |
| **Myelin** | Control - Typical AD | Hip | 4.595 | 31.627 | 27.1318 | 1.486 | -3.024 | 0.008 | 0.23595 | -15.3 |  |
|  | Typical AD - Atypical AD | Regions Combined | -2.31 | 30.658 | 32.964 | 0.898 | -2.568 | 0.036 | 0.536 | -7.2515 |  |
| **Iron** | Control - Typical AD | Regions Combined | -2.078 | 3.418 | 5.4968 | 0.633 | 3.281 | 0.006 | **0.029** | 46.62 |  |
|  |  | GFM | -3.009 | 3.203 | 6.2117 | 0.915 | 3.290 | 0.004 | **0.023** | 63.91 |  |
|  |  | GPS | -3.357 | 3.040 | 6.3976 | 0.951 | 3.530 | 0.002 | **0.015** | 71.14 |  |
|  | Control - Atypical AD | Regions Combined | -1.745 | 3.4185 | 5.164 | 0.626 | -2.788 | 0.023 | 0.081 | 40.67 |  |
|  |  | GPS | -3.611 | 3.0405 | 6.651 | 0.913 | -3.955 | 0.000 | **0.007** | 74.51 |  |
| **Microglia** | Control - Typical AD | Regions Combined | 1.393 | 4.563 | 3.171 | 0.430 | -3.237 | 0.007 | **0.038** | 36.012 |  |
|  |  | Hip | 5.166 | 9.101 | 3.934 | 0.695 | -7.430 | <0.001 | **<0.001** | 79.267 |  |
|  |  | ParaHip | 2.181 | 5.743 | 3.561 | 0.714 | -3.057 | 0.007 | **0.038** | 46.896 |  |
|  |  | Fusiform | 2.778 | 5.858 | 3.080 | 0.753 | -3.689 | 0.001 | **0.007** | 62.165 |  |
|  | Control - Atypical AD | Hip | 3.658 | 9.101 | 5.443 | 0.700 | 5.225 | <0.001 | **<0.001** | 50.308 |  |
| **Inflammation** | Control - Typical AD | Regions Combined | -0.051 | 0.865 | 0.9154 | 0.019 | 2.613 | 0.032 | 0.175 | 5.68 |  |
|  |  | ParaHip | -0.109 | 0.802 | 0.9107 | 0.034 | 3.170 | 0.005 | **0.044** | 12.71 |  |
|  |  | GFM | -0.189 | 0.716 | 0.9050 | 0.035 | 5.446 | 0.000 | **0.000** | 23.32 |  |
|  | Control - Atypical AD | ParaHip | -0.093 | 0.8018 | 0.895 | 0.035 | -2.692 | 0.021 | 0.140 | 10.96 |  |
|  |  | GFM | -0.179 | 0.7160 | 0.895 | 0.035 | -5.191 | 0.000 | **0.000** | 22.23 |  |
| Significant multiple comparison adjust values (fdr) are denoted in bold.  Hip = hippocampus, ParaHip = parahippocampal gyrus, GFM = middle frontal gyrus, GTM = middle temporal gyrus, GPS = superior parietal gyrus, Precun = precuneus, PCC = posterior cingulate cortex, OC = occipital cortex. | | | | | | | | | | | |

**Supplementary Table 5. T1w/T2w-ratio and immunohistological marker association slope estimates (β) of linear mixed regression models**

| **Variable** | **Cohort** | **Standardized β** | **SE** | **df** | **95% CI** | **t** | **p** | **p adjusted** |
| --- | --- | --- | --- | --- | --- | --- | --- | --- |
| Between-region model: T1w/T2w-ratio ~ Variable*Cohort + Age + Sex + PMD + GM + (1\|Subject) | | | | | | | | |
| **Aβ** | Control | -0.056 | 0.119 | 466.3 | -0.29 – 0.178 | -0.468 | 0.640 | 0.640 |
|  | AD | 0.233 | 0.056 | 468.2 | 0.123 – 0.343 | 4.167 | <0.001 | **<0.001** |
|  | Typical AD | 0.260 | 0.084 | 444.0 | 0.095 – 0.425 | 3.101 | 0.002 | **0.006** |
|  | Atypical AD | 0.187 | 0.081 | 454.4 | 0.029 – 0.346 | 2.328 | 0.020 | **0.043** |
| **pTau** | Control | NA | NA | NA | NA | NA | NA | NA |
|  | AD | -0.220 | 0.057 | 444.4 | -0.332 – -0.108 | -3.862 | <0.001 | **<0.001** |
|  | Typical AD | -0.309 | 0.094 | 424.5 | -0.494 – -0.124 | -3.277 | 0.001 | **0.004** |
|  | Atypical AD | -0.165 | 0.078 | 440.2 | -0.318 – -0.011 | -2.113 | 0.035 | 0.057 |
| **NfL** | Control | 0.055 | 0.093 | 440.5 | -0.127 – 0.237 | 0.595 | 0.552 | 0.640 |
|  | AD | 0.170 | 0.052 | 400.3 | 0.067 – 0.273 | 3.249 | 0.001 | **0.003** |
|  | Typical AD | 0.131 | 0.073 | 427.6 | -0.013 – 0.274 | 1.785 | 0.075 | 0.112 |
|  | Atypical AD | 0.209 | 0.072 | 426.2 | 0.068 – 0.351 | 2.908 | 0.004 | **0.010** |
| **Myelin** | Control | 0.229 | 0.08 | 375.9 | 0.071 – 0.387 | 2.850 | 0.005 | **0.009** |
|  | AD | 0.312 | 0.044 | 379.9 | 0.225 – 0.399 | 7.022 | <0.001 | **<0.001** |
|  | Typical AD | 0.283 | 0.065 | 359.3 | 0.154 – 0.411 | 4.331 | <0.001 | **<0.001** |
|  | Atypical AD | 0.302 | 0.064 | 353.2 | 0.176 – 0.427 | 4.728 | <0.001 | **<0.001** |
| **Iron** | Control | -0.359 | 0.155 | 177.4 | -0.666 – -0.052 | -2.310 | 0.022 | **0.034** |
|  | AD | 0.154 | 0.066 | 175.4 | 0.025 – 0.284 | 2.354 | 0.020 | **0.034** |
|  | Typical AD | 0.150 | 0.091 | 179.2 | -0.030 – 0.331 | 1.648 | 0.101 | 0.133 |
|  | Atypical AD | 0.164 | 0.095 | 178.9 | -0.023 – 0.350 | 1.729 | 0.086 | 0.120 |
| **Microglia** | Control | -0.090 | 0.042 | 383.9 | -0.172 – -0.008 | -2.157 | 0.032 | **0.044** |
|  | AD | -0.496 | 0.085 | 346.1 | -0.663 – -0.330 | -5.872 | <0.001 | **<0.001** |
|  | Typical AD | -0.596 | 0.164 | 359.4 | -0.920 – -0.273 | -3.626 | <0.001 | **0.002** |
|  | Atypical AD | -0.469 | 0.105 | 374.3 | -0.675 – -0.262 | -4.467 | <0.001 | **<0.001** |
| **Inflammation** | Control | -0.025 | 0.048 | 380.9 | -0.118 – 0.069 | -0.519 | 0.604 | 0.640 |
|  | AD | 0.037 | 0.079 | 367.1 | -0.118 – 0.193 | 0.470 | 0.639 | 0.640 |
|  | Typical AD | -0.073 | 0.116 | 375.2 | -0.301 – 0.154 | -0.633 | 0.527 | 0.608 |
|  | Atypical AD | 0.184 | 0.116 | 357.7 | -0.044 – 0.413 | 1.589 | 0.113 | 0.140 |
| Within-region model: T1w/T2w-ratio ~ Variable*Cohort + Age + Sex + PMD + GM + (1\|Region) + (1\|Subject) | | | | | | | | |
| **Aβ** | Control | -0.128 | 0.08 | 436.8 | -0.2857 – 0.0292 | -1.602 | 0.110 | 0.482 |
|  | AD | -0.013 | 0.042 | 458.2 | -0.0963 – 0.0703 | -0.307 | 0.759 | 0.886 |
|  | Typical AD | 0.013 | 0.059 | 433.3 | -0.1039 – 0.1290 | 0.212 | 0.832 | 0.920 |
|  | Atypical AD | -0.037 | 0.059 | 443.2 | -0.1522 – 0.0791 | -0.621 | 0.535 | 0.862 |
| **pTau** | Control | NA | NA | NA | NA | NA | NA | NA |
|  | AD | -0.006 | 0.044 | 427.6 | -0.0932 – 0.0808 | -0.140 | 0.889 | 0.901 |
|  | Typical AD | -0.051 | 0.069 | 418.2 | -0.1870 – 0.0841 | -0.746 | 0.456 | 0.862 |
|  | Atypical AD | 0.022 | 0.058 | 405.4 | -0.0915 – 0.1365 | 0.388 | 0.698 | 0.862 |
|  |  |  |  |  |  |  |  |  |
|  |  |  |  |  |  |  |  |  |
| **Continuation of supplementary Table 5.** | |  |  |  |  |  |  |  |
| **Variable** | **Cohort** | **Standardized β** | **SE** | **df** | **95% CI** | **t** | **p** | **p adjusted** |
|  |  |  |  |  |  |  |  |  |
| **NfL** | Control | -0.026 | 0.062 | 388.3 | -0.1486 – 0.0965 | -0.418 | 0.676 | 0.886 |
|  | AD | 0.038 | 0.036 | 413.8 | -0.0333 – 0.1093 | 1.047 | 0.296 | 0.591 |
|  | Typical AD | 0.044 | 0.049 | 398.4 | -0.0529 – 0.1406 | 0.891 | 0.373 | 0.862 |
|  | Atypical AD | 0.034 | 0.049 | 400.6 | -0.0635 – 0.1306 | 0.680 | 0.497 | 0.862 |
| **Myelin** | Control | 0.007 | 0.056 | 353.4 | -0.1030 – 0.1169 | 0.124 | 0.901 | 0.901 |
|  | AD | 0.045 | 0.036 | 360.5 | -0.0256 – 0.1156 | 1.254 | 0.211 | 0.590 |
|  | Typical AD | 0.031 | 0.049 | 338.4 | -0.0649 – 0.1261 | 0.631 | 0.529 | 0.862 |
|  | Atypical AD | 0.042 | 0.047 | 340.2 | -0.0501 – 0.1349 | 0.902 | 0.368 | 0.862 |
| **Iron** | Control | -0.257 | 0.119 | 162.2 | -0.4914 – -0.0231 | -2.170 | 0.031 | 0.441 |
|  | AD | 0.016 | 0.051 | 164.1 | -0.0836 – 0.1166 | 0.325 | 0.746 | 0.886 |
|  | Typical AD | 0.024 | 0.069 | 164.5 | -0.1123 – 0.1603 | 0.348 | 0.729 | 0.862 |
|  | Atypical AD | 0.011 | 0.072 | 165 | -0.1317 – 0.1542 | 0.155 | 0.877 | 0.921 |
| **Microglia** | Control | 0.044 | 0.03 | 338.9 | -0.0142 – 0.1024 | 1.487 | 0.138 | 0.482 |
|  | AD | -0.057 | 0.068 | 371.1 | -0.1896 – 0.0765 | -0.836 | 0.404 | 0.707 |
|  | Typical AD | -0.126 | 0.119 | 360.1 | -0.3597 – 0.1077 | -1.061 | 0.290 | 0.862 |
|  | Atypical AD | -0.038 | 0.079 | 343.8 | -0.1942 – 0.1183 | -0.478 | 0.633 | 0.862 |
| **Inflammation** | Control | -0.052 | 0.03 | 346.1 | -0.1114 – 0.0082 | -1.698 | 0.090 | 0.482 |
|  | AD | 0.054 | 0.05 | 352.7 | -0.0454 – 0.1527 | 1.065 | 0.288 | 0.591 |
|  | Typical AD | 0.025 | 0.073 | 334 | -0.1187 – 0.1691 | 0.345 | 0.730 | 0.862 |
|  | Atypical AD | 0.105 | 0.075 | 344.7 | -0.0418 – 0.2515 | 1.407 | 0.160 | 0.675 |
| Separate models where run for Control + AD groups and Typical + Atypical AD groups. pTau associations for the control group were set at NA due to near zero amount of variance in pTau levels. Associations estimated as significant (Kenward-Roger approximation) after fdr multiple comparison adjustment are denoted in bold. NA = Not available. | | | | | | | | |

**Supplementary Table 6. Adjusted for APOE T1w/T2w-ratio and immunohistological marker association slope estimates (β) of linear mixed regression models**

| **Variable** | **Cohort** | **Standardized β** | **SE** | **df** | **95% CI** | **t** | **p** | **p adjusted** |
| --- | --- | --- | --- | --- | --- | --- | --- | --- |
| Between-region model: T1w/T2w-ratio ~ Variable*Cohort + Age + Sex + PMD + GM + APOE (1\|Subject) | | | | | | | | |
| Aβ | Control | -0.104 | 0.123 | 448.8 | -0.345 – 0.137 | -0.851 | 0.395 | 0.477 |
|  | AD | 0.227 | 0.056 | 459.8 | 0.116 – 0.338 | 4.035 | <0.001 | **<0.001** |
|  | Typical AD | 0.256 | 0.084 | 463.1 | 0.091 – 0.422 | 3.051 | 0.002 | **0.007** |
|  | Atypical AD | 0.198 | 0.08 | 447 | 0.04 – 0.356 | 2.464 | 0.014 | **0.027** |
| pTau | Control | NA | NA | NA | NA | NA | NA | NA |
|  | AD | -0.223 | 0.059 | 440.1 | -0.34 – -0.107 | -3.759 | <0.001 | **<0.001** |
|  | Typical AD | -0.31 | 0.094 | 447.7 | -0.495 – -0.124 | -3.282 | 0.001 | **0.004** |
|  | Atypical AD | -0.172 | 0.078 | 429.5 | -0.325 – -0.019 | -2.207 | 0.028 | **0.049** |
| NfL | Control | 0.056 | 0.093 | 400.9 | -0.126 – 0.238 | 0.606 | 0.545 | 0.545 |
|  | AD | 0.179 | 0.053 | 445.5 | 0.076 – 0.283 | 3.405 | <0.001 | **0.002** |
|  | Typical AD | 0.132 | 0.073 | 434.1 | -0.012 – 0.276 | 1.804 | 0.072 | **0.094** |
|  | Atypical AD | 0.217 | 0.072 | 432.8 | 0.075 – 0.359 | 3.012 | 0.003 | **0.007** |
| Myelin | Control | 0.215 | 0.081 | 375.1 | 0.055 – 0.374 | 2.65 | 0.008 | **0.017** |
|  | AD | 0.302 | 0.046 | 370.7 | 0.213 – 0.392 | 6.638 | <0.001 | **<0.001** |
|  | Typical AD | 0.281 | 0.065 | 354.6 | 0.152 – 0.41 | 4.292 | <0.001 | **<0.001** |
|  | Atypical AD | 0.296 | 0.064 | 364.4 | 0.17 – 0.421 | 4.633 | <0.001 | **<0.001** |
| Iron | Control | -0.404 | 0.157 | 174.6 | -0.714 – -0.095 | -2.579 | 0.011 | **0.019** |
|  | AD | 0.166 | 0.065 | 185 | 0.037 – 0.295 | 2.538 | 0.012 | **0.019** |
|  | Typical AD | 0.145 | 0.091 | 184 | -0.035 – 0.325 | 1.586 | 0.114 | 0.141 |
|  | Atypical AD | 0.19 | 0.094 | 187.7 | 0.004 – 0.376 | 2.012 | 0.046 | **0.069** |
| Microglia | Control | -0.088 | 0.042 | 339.8 | -0.171 – -0.006 | -2.098 | 0.037 | 0.051 |
|  | AD | -0.502 | 0.085 | 385.4 | -0.669 – -0.334 | -5.886 | <0.001 | **<0.001** |
|  | Typical AD | -0.598 | 0.165 | 383.5 | -0.922 – -0.274 | -3.631 | <0.001 | **0.002** |
|  | Atypical AD | -0.472 | 0.105 | 366.4 | -0.679 – -0.266 | -4.503 | <0.001 | **<0.001** |
| Inflammation | Control | -0.036 | 0.048 | 361.4 | -0.13 – 0.058 | -0.749 | 0.455 | 0.49 |
|  | AD | 0.067 | 0.08 | 378.5 | -0.092 – 0.225 | 0.827 | 0.409 | 0.477 |
|  | Typical AD | -0.071 | 0.116 | 358.2 | -0.3 – 0.157 | -0.614 | 0.54 | 0.547 |
|  | Atypical AD | 0.215 | 0.116 | 381.6 | -0.014 – 0.443 | 1.843 | 0.066 | 0.092 |
| Separate models where run for Control + AD groups and Typical + Atypical AD groups. Covariate inclusion was limited on basis of best model-fit to avoid overparameterization. pTau associations for the control group were set at NA due to near zero amount of variance in pTau levels. Associations estimated as significant (Kenward-Roger approximation) after fdr multiple comparison adjustment are denoted in bold. NA = Not available. | | | | | | | | |

**Supplementary Table 7. Significant regional associations T1w/T2w-ratio with histological markers.**

| **Histological Marker** | **Group** | **Region** | **Standardized β** | **t value** | **p value** | **p adjusted** |
| --- | --- | --- | --- | --- | --- | --- |
| Regional model: T1w/T2w-ratio ~ Variable*Cohort + Variable*Region + Age + Sex + PMD + GM + (1\|Subject) | | | | | | |
| **Control and AD** |  |  |  |  |  |  |
| Aβ | Control | GFM | -0.179 | -2.07 | 0.039 | 0.338 |
| Aβ | AD | OC | 0.264 | 2.70 | 0.007 | 0.131 |
| pTau | AD | OC | 0.278 | 2.71 | 0.007 | 0.126 |
| Myelin | AD | Hip | 0.181 | 2.14 | 0.033 | 0.599 |
| Iron | Control | GPS | -0.364 | -2.72 | 0.007 | 0.073 |
| Microglia | Control | ParaHip | 0.244 | 3.14 | 0.002 | **0.029** |
| Microglia | AD | GFM | -0.168 | -2.07 | 0.039 | 0.311 |
| **Clinical AD phenotypes** | |  |  |  |  |  |
| Aβ | Typical AD | OC | 0.293 | 2.70 | 0.007 | 0.408 |
| Aβ | Atypical AD | OC | 0.241 | 2.18 | 0.030 | 0.928 |
| pTau | Typical AD | OC | 0.257 | 2.14 | 0.030 | 0.928 |
| pTau | Atypical AD | OC | 0.298 | 2.70 | 0.007 | 0.408 |
| Associations estimated as significant (Kenward-Roger approximation) after fdr multiple comparison adjustment are denoted in bold. Hip = hippocampus, ParaHip = parahippocampal gyrus, GFM = middle frontal gyrus, GPS = superior parietal gyrus, OC = occipital cortex. | | | | | | |
