# Supplementary material for: T1-weighted/T2-weighted ratio reflects microstructural changes in Alzheimer’s disease
Source: Alzheimers Res Ther. 2026 May 27;18:175. doi: 10.1186/s13195-026-02093-6 (PMC13404876; doi:10.1186/s13195-026-02093-6)
Supplement: Supplementary file 2 — Supplementary Material 2. [file 13195_2026_2093_MOESM2_ESM.docx]

**
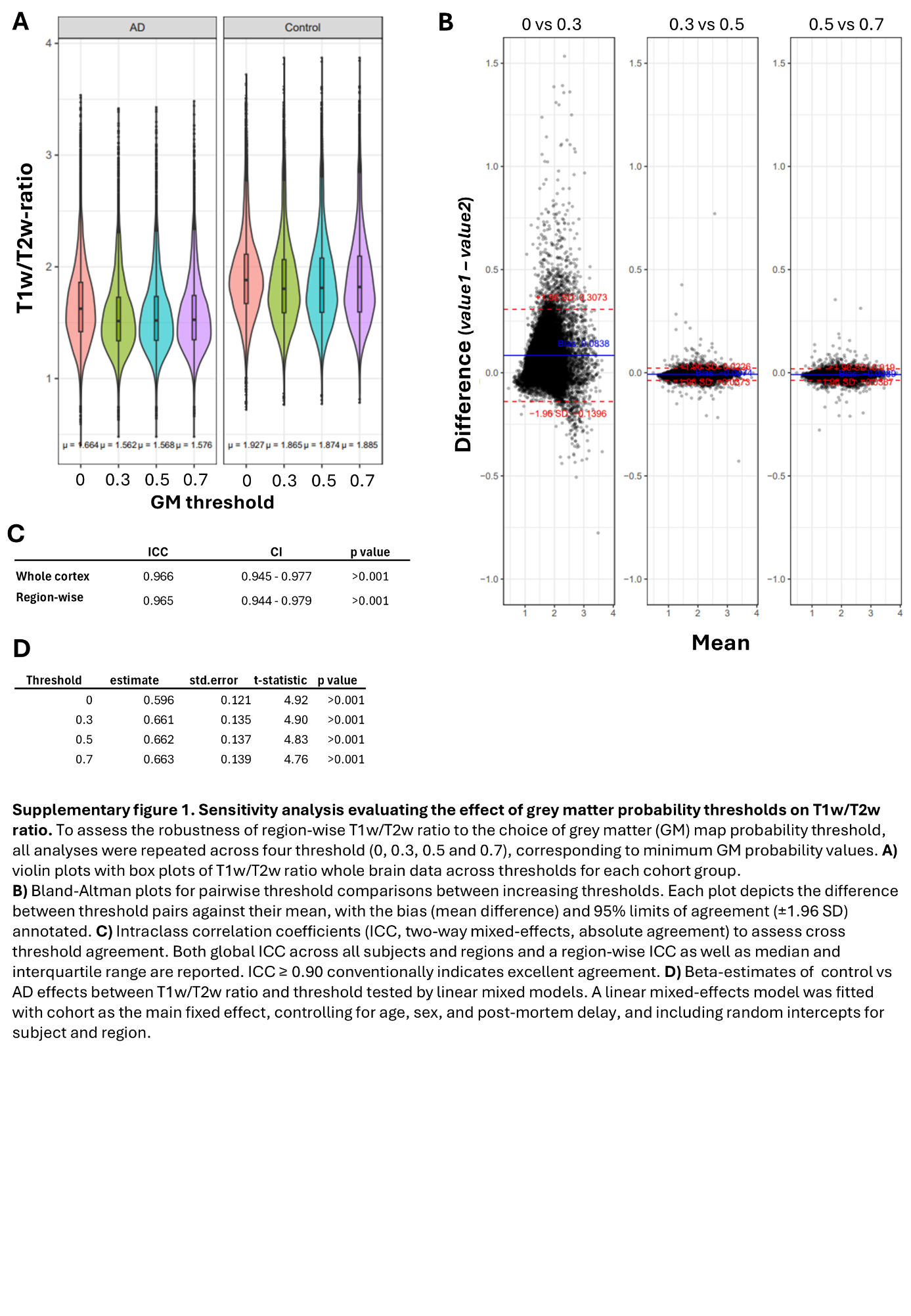
**

**Supplementary figure 1. Sensitivity analysis evaluating the effect of grey matter probability thresholds on T1w/T2w-ratio.** To assess the robustness of region-wise T1w/T2w-ratio to the choice of grey matter (GM) map probability thresholds, all analyses were repeated across four thresholds (0, 0.3, 0.5 and 0.7). A) violin plots with box plots of whole brain T1w/T2w-ratio across thresholds for each cohort group. B) Bland-Altman plots for pairwise threshold comparisons between thresholds. Each plot depicts the difference between threshold pairs against their mean, with the bias (mean difference) and 95% limits of agreement (±1.96 SD) annotated. C) Intraclass correlation coefficients (ICC, two-way mixed-effects, absolute agreement) to assess cross threshold agreement. Both global ICC across all subjects and regions and a region-wise ICC as well as median and interquartile range are reported. D) Beta-estimates of control vs AD effects between T1w/T2w-ratio and selected threshold tested by linear mixed models. A linear mixed-effects model was fitted with cohort as the main fixed effect, controlling for age, sex, and postmortem delay, and including random intercepts for subject and region.

**
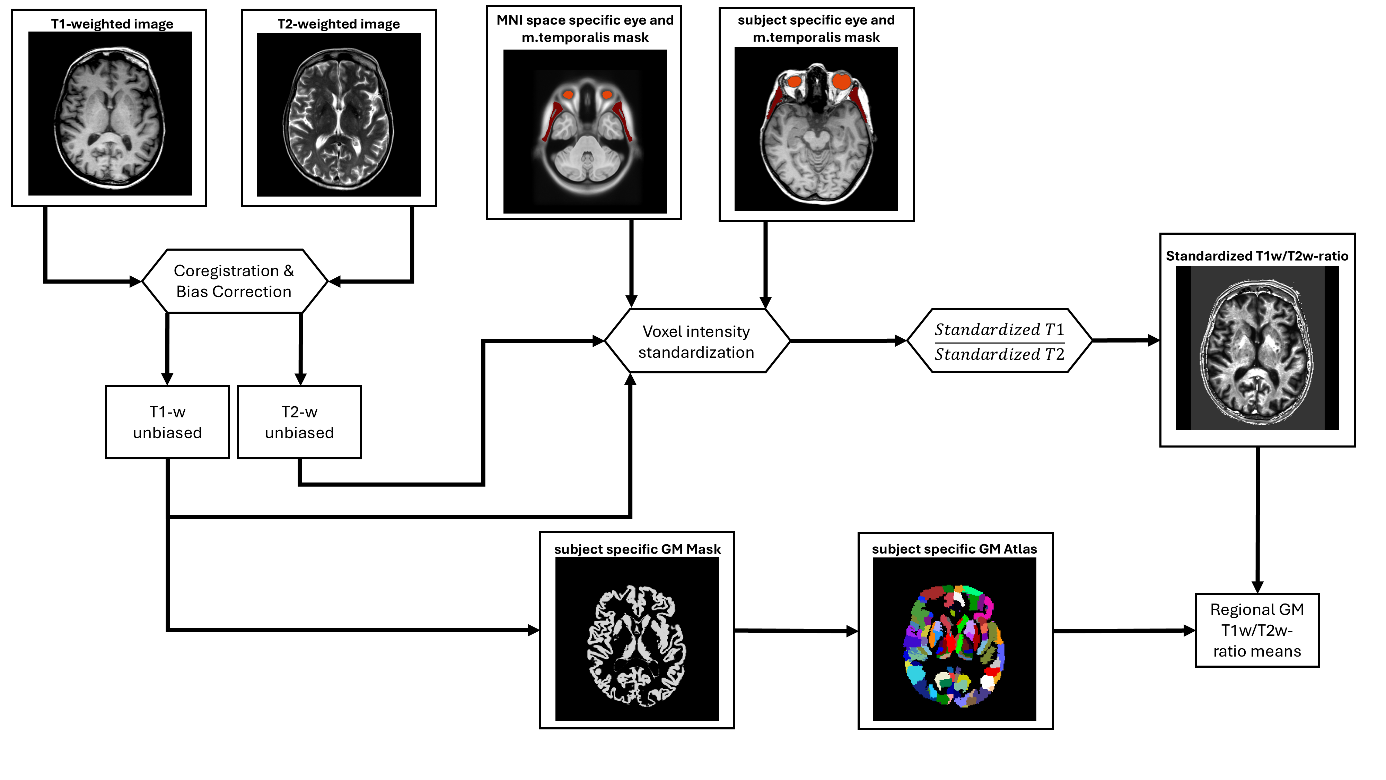
**

**Supplementary Figure 2. T1w/T2w-ratio processing pipeline.** T1-weighted and T2-weighted images were coregistered to the same subject space and bias corrected. Grey matter (GM) segmentation and brainnetome atlas parcellation were performed on T1-weighted images to obtain a subject specific GM atlas. Voxel intensity of T1-weighted and T2-weighted images was standardized using methodology described by Ganzetti *et al*. (2014) where modal voxel intensity of the eye and m.temporalis in both the IBM152 template brain and subject brain was used in a linear transformation model. Standardized T1-weighted images were divided by T2-weighted images, thresholded for extreme outliers (voxel intensity >6), resulting in a standardized T1w/T2w-ratio. Subject specific GM atlas masks were used to extract mean regional GM T1w/T2w-ratio values. All processes were performed in SPM12.


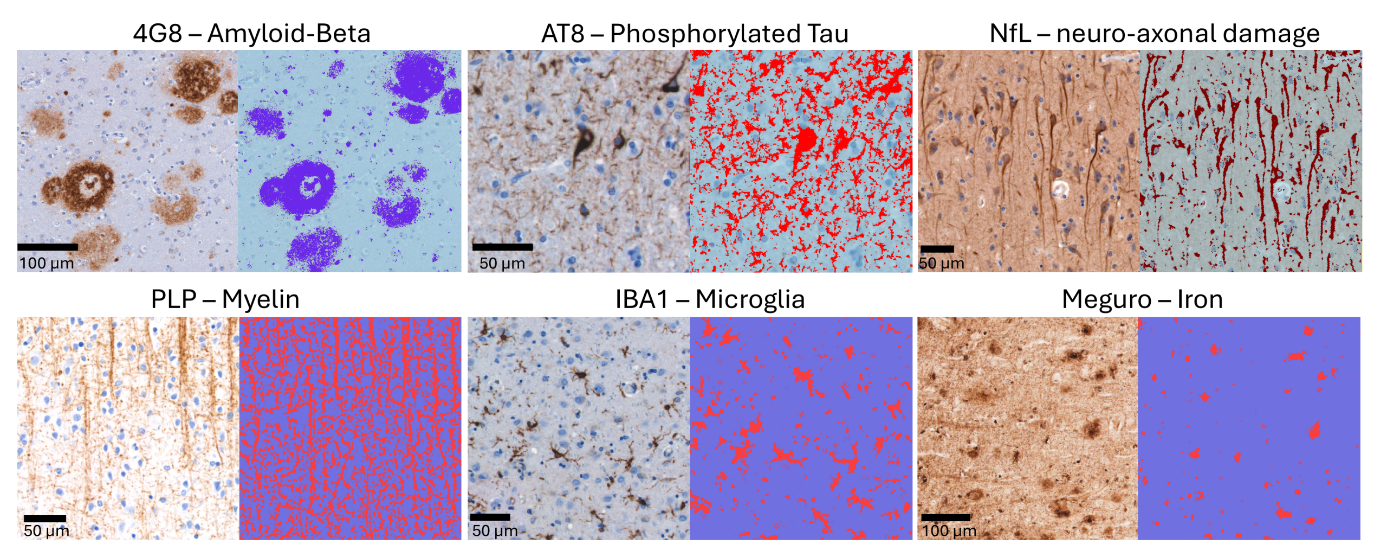


**Supplementary Figure 3. Histological marker images for microstructural change.** All markers are named first by their relevant staining method followed by the biological aspect that was targeted. For all markers left images display the histology, supplemented by a scale bar in microns, and right images display the digital quantification results of the pixel classifier built in Qupath.


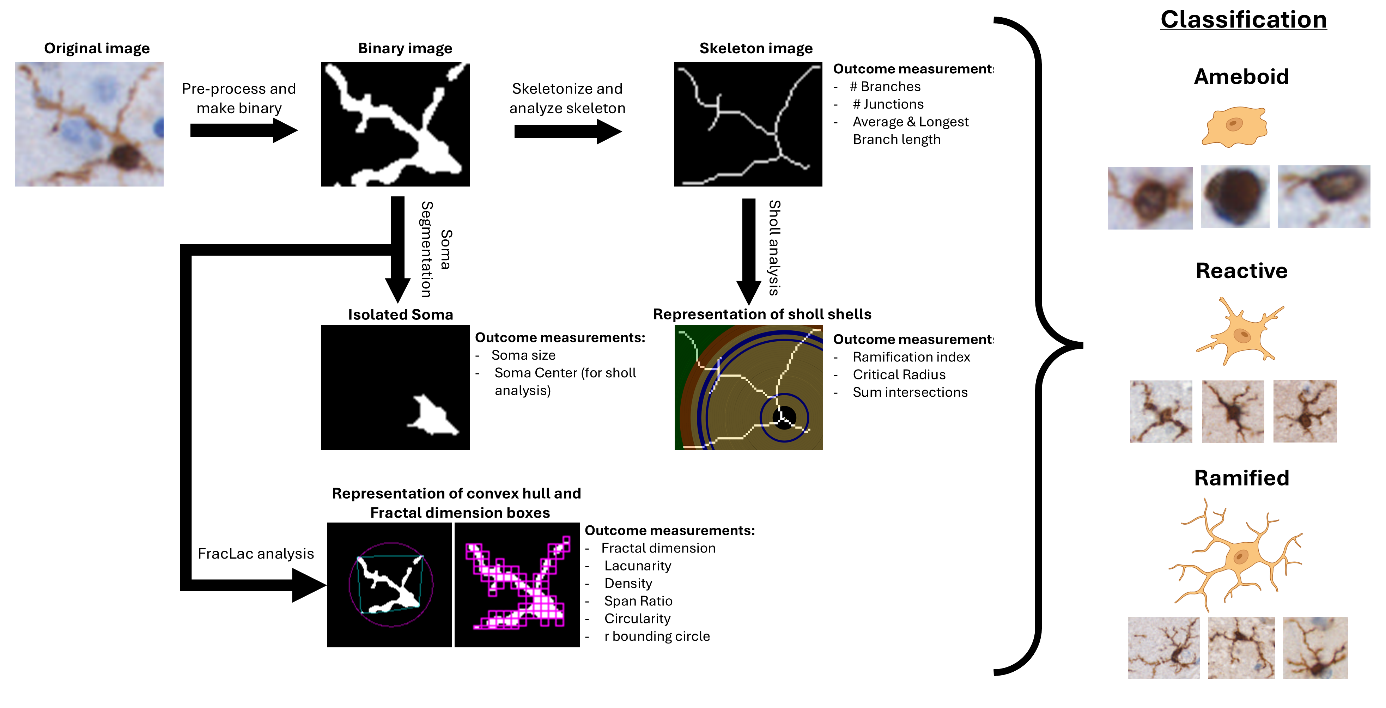


**Supplementary Figure 4. Automized microglia morphology processing pipeline.** Individual microglia images were extracted using a combination of pixel and object classifiers build in Qupath. Microglia were first binarized followed by a skeletonization, which includes a branch connecting step to avoid potentially disconnected paths caused by processing. Skeletonized images were then used for skeleton, branch and Sholl analysis. Cell Soma was segmented and analyzed using thresholding and the distance transform watershed algorithm. Additionally, soma center was used for input into the Sholl analysis. The FracLac package was used on the binarized images to assess fractal dimension, lacunarity and additional geometric measurements. These parameters were used to manually classify microglia in ameboid morphology and to use a k-means clustering algorithm for classication into reactive and ramified morphologies.


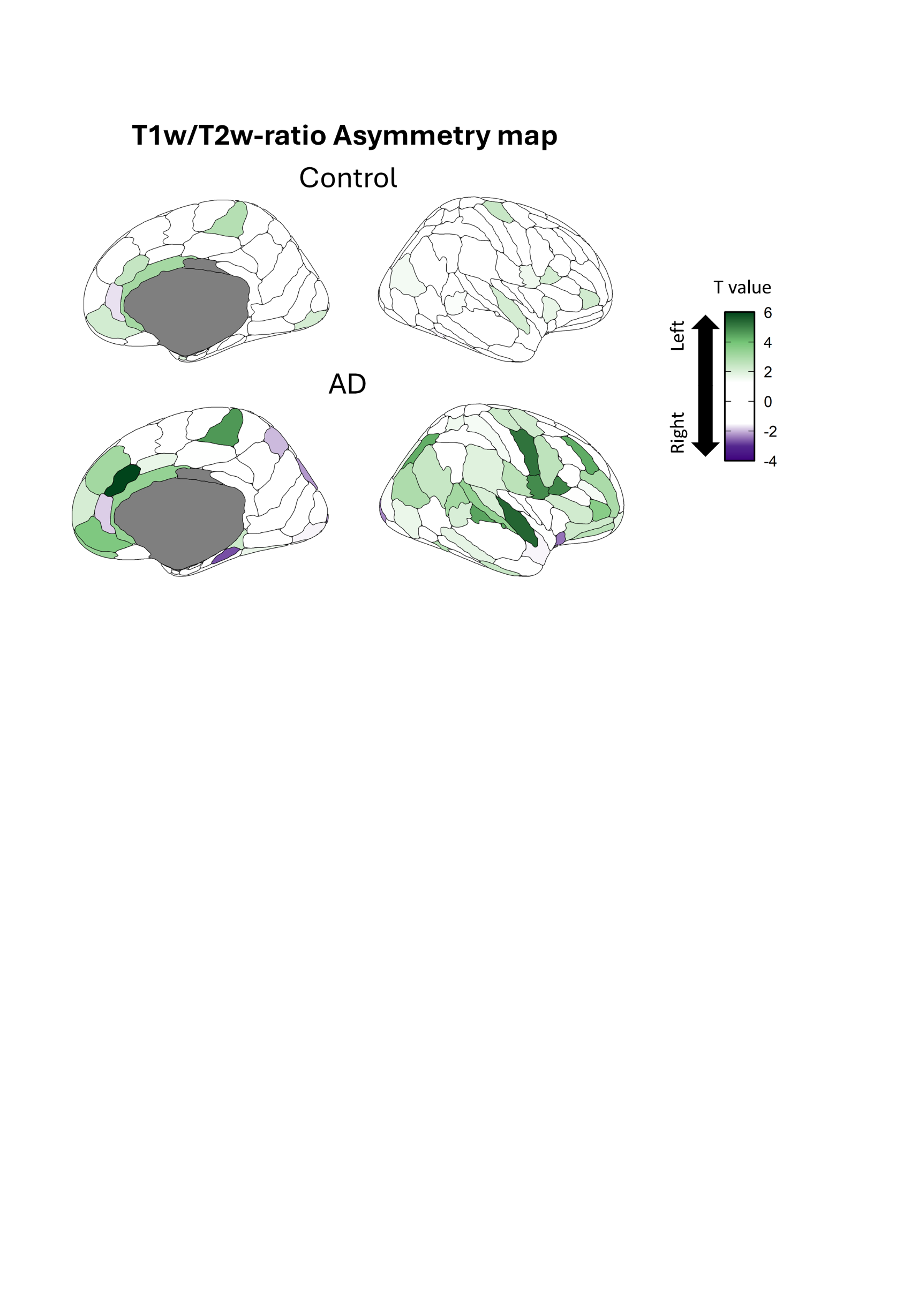


**Supplementary Figure 5. T1w/T2w-ratio Asymmetry map for Controls and AD.** Difference between left and right hemisphere is displayed in t value, green displaying a higher T1w/T2w-ratio in the left hemisphere and purple representing a higher T1w/T2w-ratio in the right hemisphere. A predominant higher left T1w/T2w-ratio can be seen in AD, especially in frontal and lateral parietal and temporal regions, while the differences are low in controls.


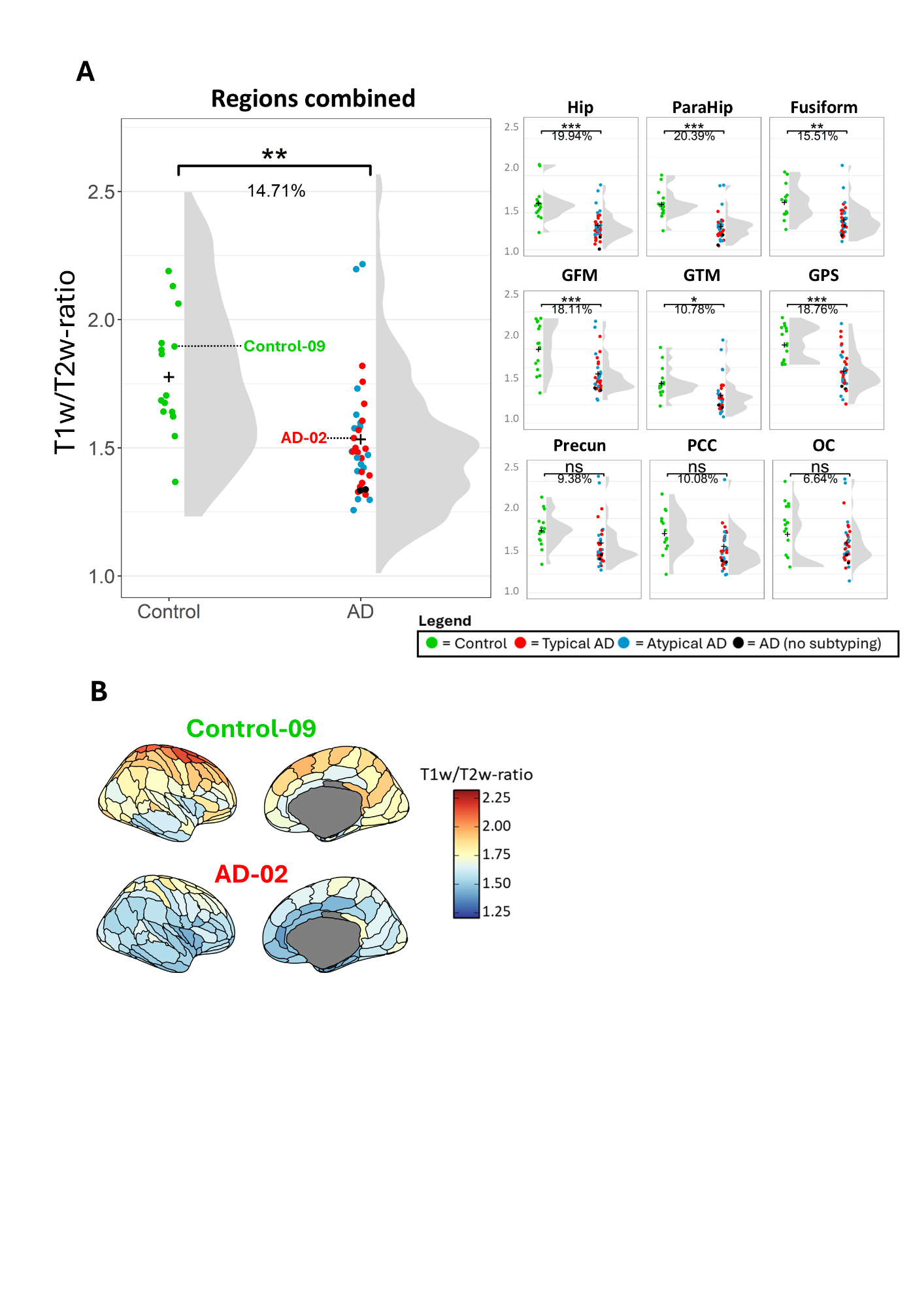


**Supplementary Figure 6. T1w/T2w-ratio distribution and contrast between groups of IHC subset regions.** A) Regions combined and regional distribution of T1w/T2w-ratios in controls, AD, and AD clinical phenotypes. A global reduction in the T1w/T2w-ratio is observed in AD (14.7%), with accompanying statistically significant regional reductions. Points represent mean values for each subject; raincloud plots illustrate the overall distribution of regional datapoints. B) Single subject T1w/T2w-ratio map for Control-09 and AD-02 cases to illustrate raw whole brain maps (all atlas regions) * = *p* ≤ 0.05, ** = *p* ≤ 0.01, *** = *p* ≤ 0.001, ns = not significant, + = estimated marginal mean. Hip = hippocampus, ParaHip = parahippocampal gyrus, GFM = middle frontal gyrus, GTM = middle temporal gyrus, GPS = superior parietal gyrus, Precun = precuneus, PCC = posterior cingulate cortex, OC = occipital cortex.


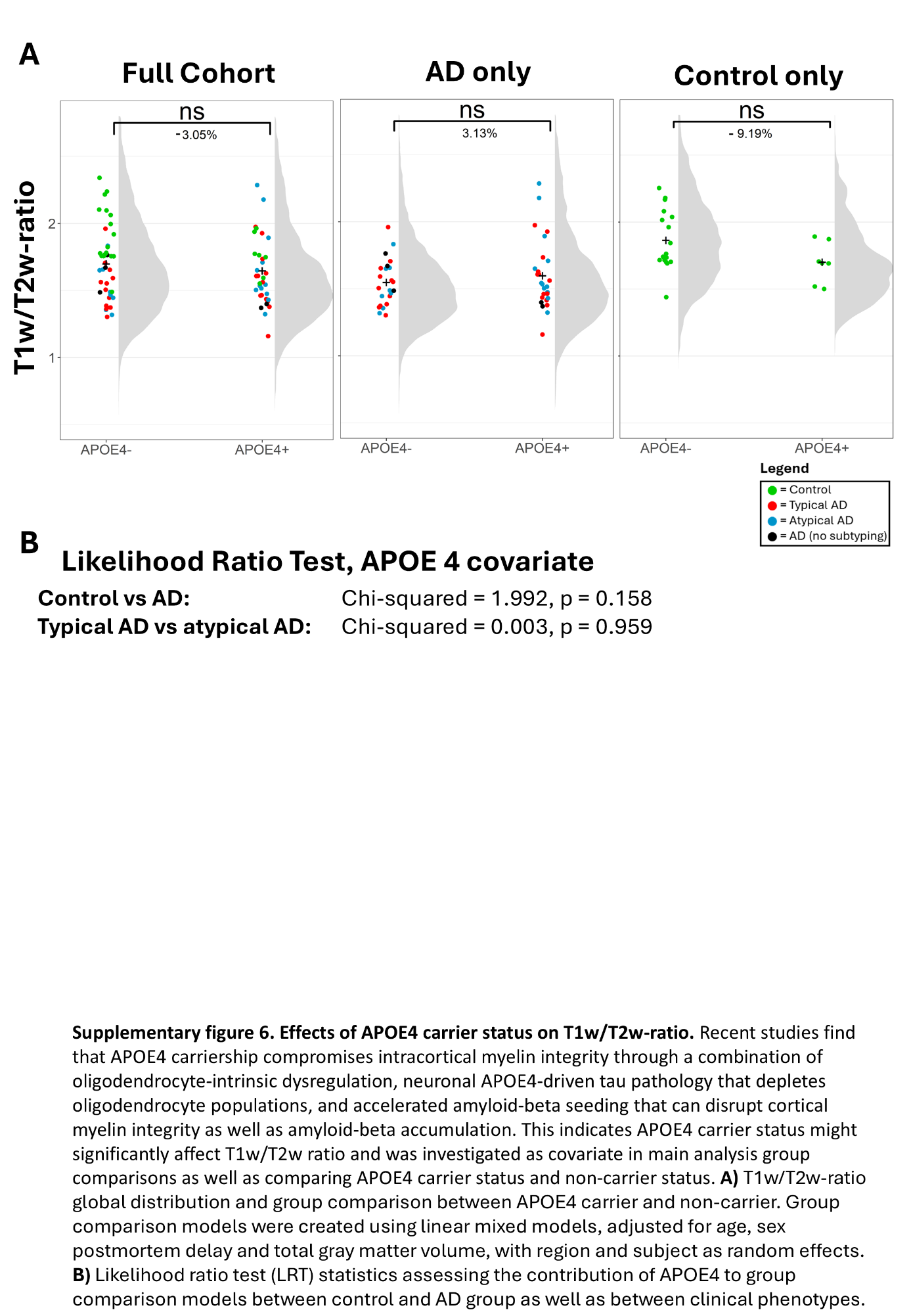


**Supplementary figure 7. Effects of APOE4 carrier status on T1w/T2w-ratio.** A) T1w/T2w-ratio global distribution and group comparison between APOE4 carrier and non-carrier. Group comparison models were created using linear mixed models, adjusted for age, sex, postmortem delay and total grey matter volume, with region and subject as random effects. B) Likelihood ratio test (LRT) statistics assessing the contribution of APOE4 as covariate to group comparison models between control and AD groups as well as between clinical phenotypes.

**
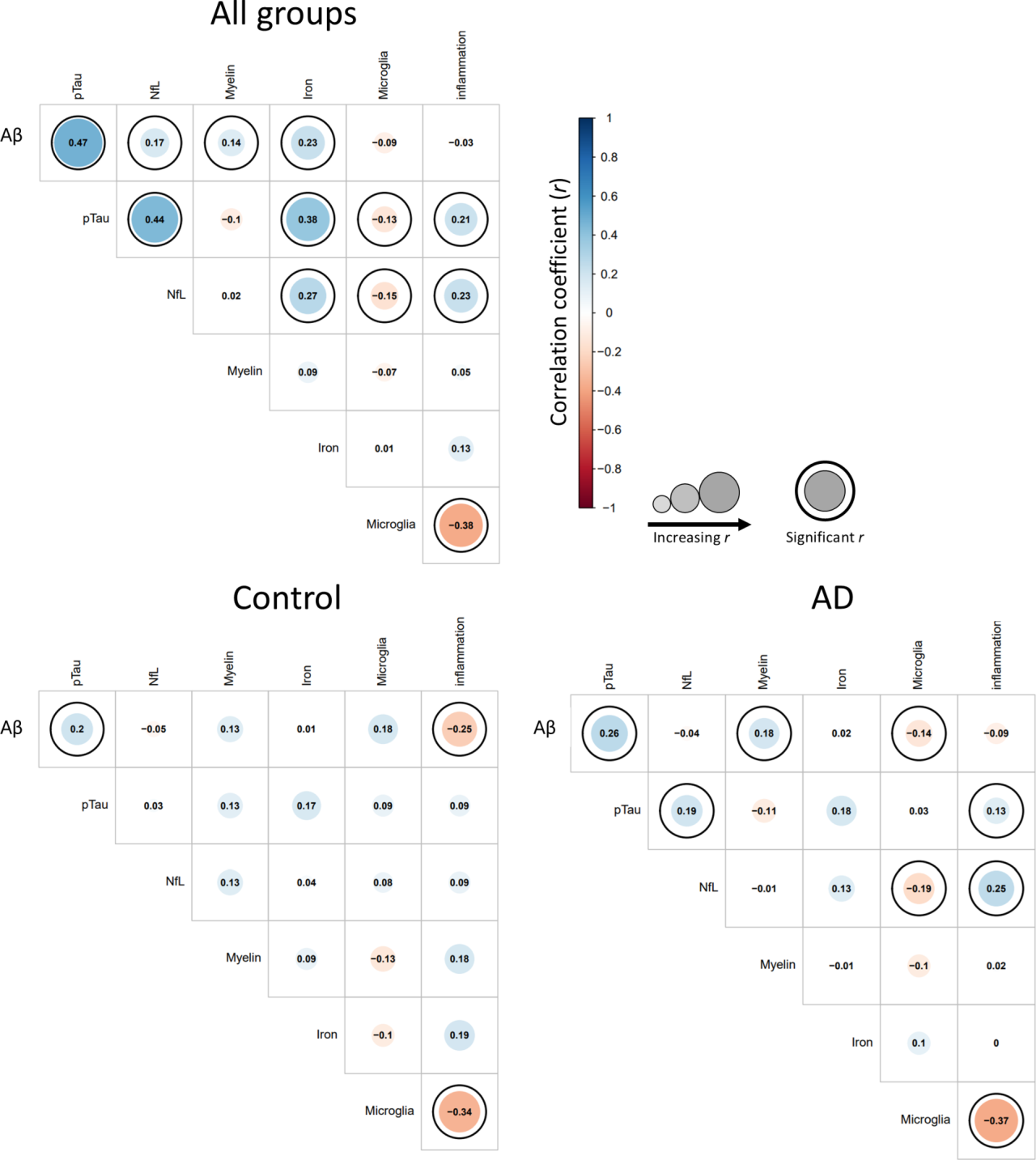
**

**Supplementary Figure 8. Correlations between histological markers for microstructural changes.** Blue signifies a positive correlation while red signifies a negative correlation. Increasing circle sizes represent a greater correlation coefficient, with a bold second encompassing circle indicating a significant correlation. Correlations were assessed for the entire cohort as well as control and AD groups separately.


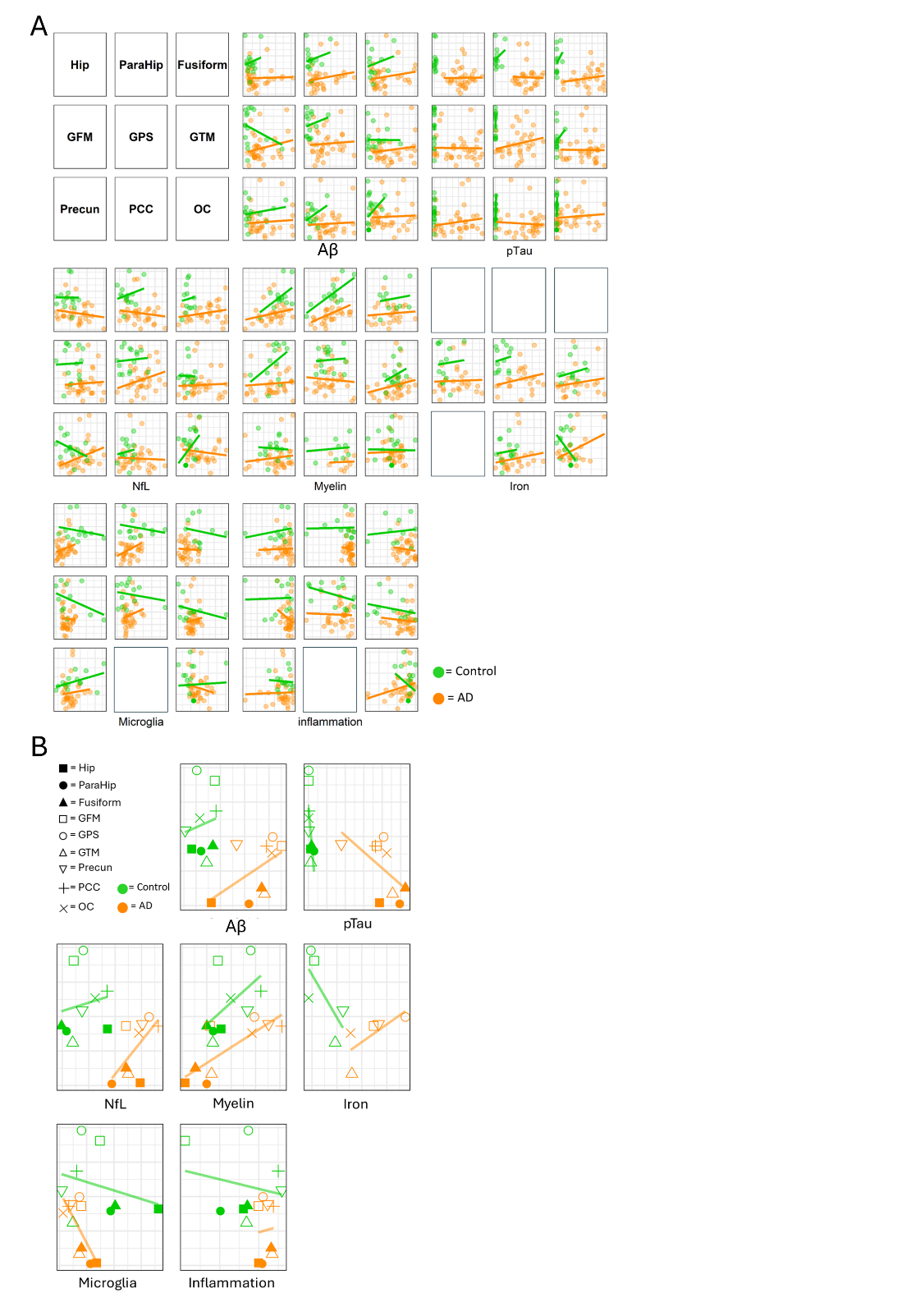


**Supplementary Figure 9. T1w/T2w-ratio and histological regional and region mean scatter plots.** A) for each histological marker regional scatter plots are displayed for both control and AD groups with a roster order as displayed in the top-left corner. B) T1w/T2w-ratio and histological marker mean value scatterplots are plotted for each marker and for both control and AD groups with a shape differentiation for each region. Hip = hippocampus, ParaHip = parahippocampal gyrus, GFM = middle frontal gyrus, GTM = middle temporal gyrus, GPS = superior parietal gyrus, Precun = precuneus, PCC = posterior cingulate cortex, OC = occipital cortex.

**
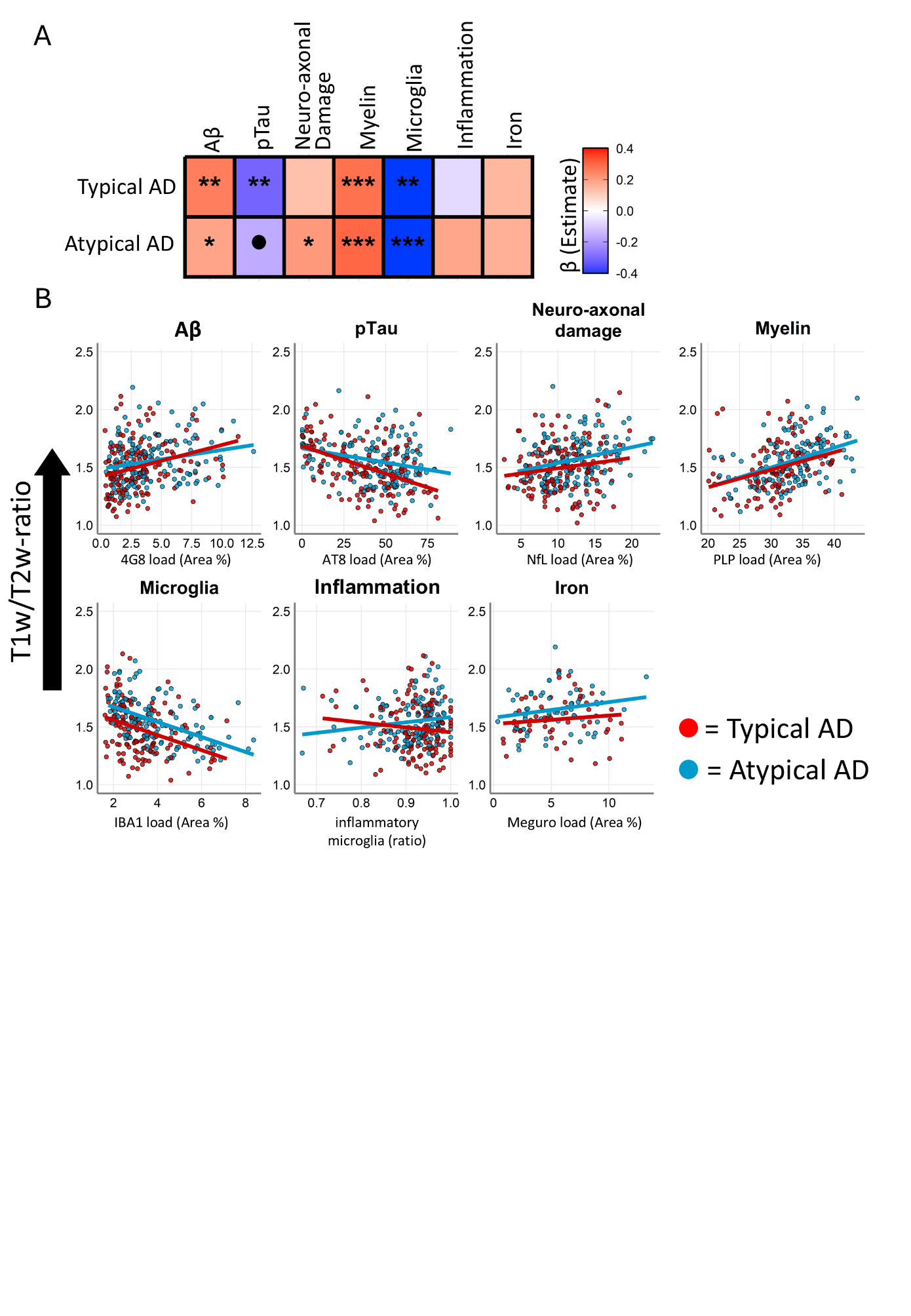
**

**Supplementary Figure 10. Associations between T1w/T2w-ratio and histological markers.** A) association (β estimate; standardized) for each histological marker in both typical and atypical AD groups, showing a strong pTau association in typical AD which is absent in atypical AD and vice versa for neuro-axonal damage. B) Distribution of T1w/T2w-plotted against histological markers for both typical and atypical AD groups. * = p ≤ 0.05, ** = p ≤ 0.01, *** = p ≤ 0.001, • = non-fdr adjusted p ≤ 0.05.
